# The European regulatory system for plant protection products—cause of a “Silent Spring” or highly advanced and protective?

**DOI:** 10.1093/inteam/vjae007

**Published:** 2025-01-06

**Authors:** Carola Schriever, Bernhard Jene, Herbert Resseler, Robert Spatz, Robin Sur, Arnd Weyers, Mark Winter

**Affiliations:** BASF SE, Agricultural Solutions, Environmental Fate, Limburgerhof, Germany; BASF SE, Agricultural Solutions, Environmental Fate, Limburgerhof, Germany; Syngenta Agro GmbH, Frankfurt am Main, Germany; Syngenta Agro GmbH, Frankfurt am Main, Germany; Bayer AG, Crop Science, Environmental Safety, Monheim am Rhein, Germany; Bayer AG, Crop Science, Environmental Safety, Monheim am Rhein, Germany; Industrieverband Agrar e. V. (IVA), Wissenschaft und Innovation, Frankfurt am Main, Germany

**Keywords:** pesticide authorization, environmental risk assessment, risk benefit analysis, biodiversity, risk mitigation

## Abstract

Current publications that are shaping public perception repeatedly claim that residues of plant protection products (PPP) in the environment demonstrate gaps in assessing the exposure and effects of PPP, allegedly revealing the inability of the European regulatory system to prevent environmental contamination and damage such as biodiversity decline. The hypothesis is that environmental risk assessments rely on inappropriate predictive models that underestimate exposure and do not explicitly account for the impact of combinations of environmental stressors and physiological differences in stress responses. This article puts this criticism into context to allow for a more balanced evaluation of the European regulatory system for PPP. There is broad consensus that the decline in biodiversity is real. This article analyzed current literature for causes of this decline and of chemical contamination. The main drivers identified were land use changes and structural uniformity of agricultural landscapes or multiple contaminants emitted by various sources such as wastewater discharge systems. Comparing measured environmental concentrations from published monitoring studies with exposure predictions from the regulatory risk assessment reveals only slight occasional exceedances for a few environmental scenarios and compounds. Therefore, the call for greater conservatism in the European authorization process for PPPs will not lead to an improvement in the environmental situation. We suggest enhancing landscape diversity through the European Union Common Agricultural Policy and reducing contamination from wastewater and farmyard effluents. The current regulatory risk management toolbox should be expanded to include flexible localized mitigation measures and treatment options to reduce applied amounts and off-target exposure.

## Teaser

**Key points**

- This article puts recent criticisms of the European regulatory system for plant protection into context to allow a more balanced assessment of the European authorisation process.

## Introduction

### Plant protection product approval procedure

Synthetic and biological plant protection products (PPPs; pesticides) are used in crop production to control pests and weeds to safeguard yields. Due to the potential risks associated with pesticides, the use of PPPs is subject to extensive legal regulations. Regulatory systems for the assessment of PPPs and their active substances have continuously developed over the last few decades, particularly in Europe. European Union (EU) pesticide laws are considered the strictest in the world (EC, n.d.). In the European Community, an active substance application dossier is peer-reviewed by the European Food Safety Authority (EFSA) together with the national regulatory authorities of 27 member states (EFSA, n.d.-a). For the subsequent authorization of any PPP containing the active substance, the respective application dossier must pass further reviews at zonal and national levels.

### Constantly evolving regulatory system for PPPs

Identified knowledge gaps trigger new or modified regulatory data requirements and risk assessments by EFSA. Since 2009, EFSA has published 11 guidance documents relating to pesticide risk assessment (EFSA, n.d.-b). A recent evaluation commissioned by EFSA (Oltmans et al., 2023) compared data requirements and evaluation criteria in several EU regulatory areas (pesticides, biocides, food and feed additives, human and veterinary medicinal products, cosmetics, and food additives and flavorings). For ecotoxicity, the number of data requirements, assessment criteria, and the degree of scrutiny in the risk assessment for pesticides was higher than for any other regulation area. Consequently, the environmental risks associated with the use of pesticides have declined considerably over recent decades, which is confirmed by declining values of several environmental and human risk indicators, particularly during the last 10 years (see, e.g., Pesticide Trends Database Explorer hosted by the Julius-Kühn-Institut [JKI, 2022]). For example, SYNOPS, developed at the JKI, quantifies the risk decline by linking the amount of PPP applied, the environmental exposure and the toxicity for aquatic and terrestrial organisms (Nause et al., 2021; Strassemeyer et al., 2017; Strassemeyer & Gutsche, 2010).

This contrasts with the claims of some authors (e.g., Liess et al., 2021; Schäffer et al., 2018, 2019; Topping et al., 2020) that detections of PPPs in the environment and knowledge gaps regarding effects would demonstrate the inability of the regulatory system to prevent environmental damage. During the same time course, considerable improvements have been made in analytical technology, allowing lower detection limits to be reached.

### Natural versus synthetic pesticides

Organic farming is often proposed as the straightforward and easy solution to increasing biodiversity and avoiding environmental damage (EC, 2020). However, even organic farming depends on the use of pesticides, in particular, in fruit and vegetable production. Natural compounds such as copper, sulfur, spinosad, and pyrethrin products are applied on both organic and conventional farms. Such substances act as “chemicals” in the same way as synthetic substances (Ames et al., 1990; Burandt et al., 2024), and may have side effects in agricultural fields or the adjacent landscape, regardless of the farm management system used.

Scientifically, it can be questioned whether the origin of a compound is more relevant than its effect. Meemken and Qaim (2018) propose that “Ideological barriers between supporters and opponents of organic agriculture need to be overcome to pave the way for developing and implementing more sustainable forms of farming” (p. 57). For the area of human toxicology, the German Federal Institute for Risk Assessment requests more evidence-based arguments in the risk-benefit assessment applied to chemicals, instead of “Promoting unjustified beliefs and anxieties…” (Herzler et al., 2021, p. 2599).

### Misconceptions about the regulatory authorization process

Several recent papers criticize the EU regulatory process for PPPs for not being sufficiently protective of the environment (e.g., Liess et al., 2021; Müller & Hitzfeld, 2020; Schäffer et al., 2018). Schäffer et al. (2018) was published under the auspices of “Leopoldina,” the German National Academy of Sciences, as a discussion paper and received some attention in media articles. Many of its claims about alleged shortcomings of the regulation of PPPs are not new and were also reflected in other publications, but here we refer to it as one prominent example of those claims.

The above articles propose changes to the authorization procedure, because some monitoring results exceed regulatory acceptable concentrations (RACs). It is important to identify the factors driving such instances. Exposure pathways related to the actual application of a product are well understood and regulated by the PPP authorization process, including the option to impose risk mitigation measures (e.g., buffer strips, drift reduction nozzles). However, other exposure pathways such as accidental spillages or wastewater effluents into water bodies (point sources) are covered by other legislative areas and must be addressed outside of the authorization process.

It is the purpose of our article to discuss and correct some common misconceptions about the adequacy and protectiveness of the EU regulatory system. The structure of this article follows broader claims of (i) impacts on biodiversity, (ii) deficiencies in ecotoxicological testing and assessment, (iii) measured concentrations in the environment exceeding those predicted in regulatory risk assessment, and (iv) inappropriateness of the current authorization procedure. The article closes with some indications on how to mitigate the environmental impact of agriculture and plant protection measures.

## Biodiversity and PPPs

### Biodiversity decline

Schäffer et al. (2018) set the frame for their article by stating that a variety of stressors (climate change, major changes to global nutrient cycles, habitat destruction, pesticides, etc.) have caused a dramatic decrease in biodiversity in Germany and around the world. This is referred to as a “mass extinction.” They suggest PPPs are a major driver of global and German biodiversity decline, quoting Rachel Carson’s 1962 book *Silent Spring* and then narrowing down their assessment of biodiversity threats exclusively to pesticides.

Rockström et al. (2009a) defined nine “planetary boundaries within which … humanity can operate safely” (Abstract) and estimate that the boundary of the rate of biodiversity loss has already been transgressed, which may lead to large-scale environmental changes. Schäffer et al. (2018) refer to the planetary boundary concept and claim that “pesticide contamination plays a significant role in the concept of planetary boundaries” (p. 6), quoting Rockström et al. (2009b), Persson et al. (2013), and Steffen et al. (2015). However, pesticides are mentioned in only one of these articles (Rockström et al., 2009b), and only as an example for persistent organic pollutants—a category which does not include any of the PPPs used in the EU.

Schäffer et al. (2018) claim that “the present use of pesticides has a significant adverse impact on ecosystems and biodiversity” (p. 17) and refer to Dirzo et al. (2014) when stating that invertebrate population sizes have declined by some 45% worldwide, alongside a dramatic decline in species numbers. Dirzo et al. (2014) do not mention pesticides and biocides as possible causes at all, but state: “The long-established major proximate drivers of wildlife population decline and extinction in terrestrial ecosystems—namely, overexploitation, habitat destruction, and impacts from invasive species—remain pervasive. None of these major drivers have been effectively mitigated at the global scale” (p. 403).

Schäffer et al. (2018) state that bird decline can be attributed to the use of imidacloprid based on a correlation (not causality) found by Hallmann et al. (2014). However, the reported bird decline in the different study sites was practically identical before and after the introduction of imidacloprid and therefore indicates trends related to land use changes in those sites.

There is broad consensus that biodiversity decline is multifactorial. Other publications under the auspices of Leopoldina (Anton et al., 2018, 2020) concluded that the causes “are predominantly due to the interaction of changes in the intensity of use, ground cover, and structure of the agricultural landscape” (Anton et al., 2018, p. 9). Causes outside the agricultural landscape are urbanization, land consolidation, and river straightening, among others.

Boyes et al. (2021) clearly demonstrate the detrimental effect of streetlights on insects. Baranov et al. (2020), for example, studied the headwaters of a watercourse located entirely in a nature reserve surrounded by forests in a low mountain range in Hesse (Germany) over a period of 42 years. In the absence of common anthropogenic stressors (including PPPs), they found a decline in insect abundance of over 80% during the study period. According to these authors, climate change is of decisive importance.

Global biodiversity decline is real. However, recent meta studies analyzing more than 4,000 studies again confirmed that biodiversity decline is multifactorial, and the three main driving factors for this decline are land use change, resource exploitation, and climate change (Leenhardt et al., 2022; Rumohr et al., 2023).

### Biodiversity and modern agriculture

Schäffer et al. (2018) suggest reducing the intensity of land use by restricting PPPs. This means a major reduction in land use efficiency for food production on the agricultural area currently used (EP et al., 2019). It will lead to additional land use elsewhere, which conflicts with the aim of conserving nonagricultural habitats (Bystricky et al., 2020; Gabriel et al., 2013; Hodgson et al., 2010; Noleppa, 2016; Smith et al., 2019 ). To cover the shortfall in food production in EU, even more food would have to be imported (Zabel et al., 2019). For example, the self-sufficiency level of Germany is currently at only 81% (BLE, n.d.) (see online supplementary material Table S1). Fuchs et al. (2020) conclude that Europe would then be exporting environmental damage to other countries. Recently, Balmford (2021) and Grass et al. (2021) reviewed which combinations of land sharing vs. land sparing approaches (i.e., essentially in-field vs. off-field biodiversity measures) are optimal for meeting the need for both undisturbed habitats and sufficient food production. They recommend focusing production in high-yield areas complemented with some land sharing approaches, improvements of crop diversity, and marginal habitats (hedgerows, flower strips).

There is robust evidence that effective coexistence of desired biodiversity levels and modern agriculture is possible (Hinsley et al., 2010; Pywell et al., 2004, 2007; Sutton et al., 2017), even when using only a small part (1%–5%) of the agricultural area for conservation purposes (Redhead et al., 2022). Hedgerows and flowering areas can readily be established in any form of agriculture, not exclusively on organic farms. Many examples show that threatened species (e.g., skylark, corn bunting, ortolan, rock bunting) do occur on or close to conventionally managed land (with the use of PPPs). The most important established measures for the protection of agricultural species focus on the availability and quality of such margin habitats (hedges, field margins, small water bodies, riparian strips etc.; see, e.g., University of Cambridge, 2020). Some measures can be implemented on the farmland itself (lark window, wide row, undersowing, etc.) and may also include managing a certain proportion of the fields less intensively.

### Science-based evaluation of risks and benefits of plant protection measures

The impact of plant protection measures on biodiversity has to be considered within the agricultural context and not in isolation. Risks and benefits of different approaches to achieving the same goal must be gauged in a similar way, as proposed by Hodgson et al. (2010) and Balmford (2021). Reducing noncrop plants with a broad-spectrum herbicide will have indirect effects on other organisms dependent on these noncrop plants, either as habitats or food. However, if no herbicide is applied, alternative methods to reduce weed competition are needed, such as the physically destructive techniques of ploughing and harrowing. According to results from Koning et al. (2019), the effect of mechanical weed management is similar to the effect of the herbicide, i.e., reduced abundance of noncrop plants and therefore of organisms living on these plants. In addition, the mechanical measures have unwanted side effects, too, for example, on ground nesting birds (Lokemoen & Beiser, 1997), migrating amphibians (Berger et al., 2011) or soil organisms (Giannitsopoulos et al., 2019), runoff, and soil erosion (Alix et al., 2017). When deciding on the use of a synthetic PPP, looking at its impacts in isolation and ignoring the impacts of the alternative methods is therefore an unsuitable approach to increase biodiversity and safeguard soil health.

## Potential gaps in assessing the effects of PPPs on biota

### Differences in species sensitivity

The usual approach for assessing the hazards from chemicals in the controlled laboratory environment is testing a defined selection of surrogate species. Remaining uncertainties, including differences in species sensitivity, are considered by means of additional safety factors anchored in the authorization procedure. For animal welfare reasons, it is neither feasible nor desirable to test many more species, particularly not more vertebrates, unless essential for the risk assessment.

Schäffer et al. (2018) criticize that the differences in sensitivity between species are not reflected by the safety factors, e.g., for earthworms or (in relation to neonicotinoids) for aquatic species. However, for earthworms, extensive comparisons between laboratory and field studies (which includes assessment of multiple species) have shown the risk assessment approach used in the EU for over 20 years to be sufficiently protective (Christl et al., 2016). Concerning neonicotinoids, it is well known that the crustacean *Daphnia magna*, although the typical surrogate species for aquatic invertebrates, is insensitive to this group of insecticides. However, tests for insecticides such as neonicotinoids on additional species (e.g., aquatic life stages of the insect *Chironomus riparius*), have been mandatory for the regulatory risk assessment of insecticides in the EU for about 20 years. For aquatic organisms, comparisons between laboratory and mesocosm studies have also confirmed the protectiveness of the approach based on the testing of standard species (Brock et al., 2006, 2016; Maltby et al, 2005; Rico et al., 2019). Similarly, data evaluations have shown that ecotoxicological data for fish are generally sufficiently protective for the aquatic life stages of amphibians (Weltje et al., 2013).

In summary, the above-mentioned examples from Schäffer et al. (2018) do not show that differences in species sensitivity are insufficiently considered by the EU approval system of PPPs. There is therefore no reason for demanding a major revision of the EU regulatory system. Rather, the examples illustrate that the regulatory system is constantly evolving and dynamically adapting to new scientific developments. Overall, test requirements have increased in the EU where data gaps were apparent (e.g., bees, soil organisms, aquatic macrophytes), but were also reduced in a few cases for vertebrates, mainly for animal welfare reasons. The 3R principles (reduction, replacement, refinement) (Russell & Burch, 1992) aim to reduce animal testing through, e.g., ecological modeling or new approach methodologies such as quantitative structure–activity relationship (QSAR), omics, and artificial intelligence (Tarazona et al., 2022).

### Combination of stressors

Schäffer et al. (2018) state that “Tank mixtures, sequential exposure and total loading are given inadequate attention” (p. 25). Still, a large-scale monitoring program at EU level indicates that mixture toxicity in European surface waters is largely driven by a few legacy chemicals and most sites are not at risk, even taking account of cumulative exposure (Rodea-Palomares et al., 2022). If ubiquitous priority substances—which are already regulated as strictly as possible—were excluded, only 3% of the EU surface water bodies would fail to achieve good chemical status (EEA et al., 2012). As an example of a previously polluted large watershed, toxic pressure in the River Rhine (measured in bioassays and not by detection of chemicals) in the year 2000 was already very low but has decreased further yet (IKSR, 2021; RIVM (National Institute for Public Health and the Environment), 2010).

Without providing more details, Schäffer et al. (2018) claim that only single active substances and representative formulations are evaluated within the risk assessment and that cumulative effects were inadequately addressed. However, this is only true for the centralized EU-wide active substance assessment. After this European approval of the active substance(s), every formulation or product needs to be assessed at zonal and national levels to obtain an authorization, including cumulative assessments when products contain more than one active substance (EC, 2013a, 2013b; EPC, 2009). Thus, although mixture assessment schemes currently do not include spray series and landscape level assessments, the broad claim that only single active substances are addressed in the current environmental risk assessment for pesticides is incorrect.

### Additional stressors

Schäffer et al. (2018) state that “the effects of pesticides, at concentrations classified as safe in the authorization process, are detectable in numerous natural landscapes, even at very low concentrations” (p. 27) when using the species at risk (SPEAR) indicator system, which can be applied to benthic macroinvertebrate data (Liess & von der Ohe, 2005). The authors suggest that “the cause of these deficient risk estimations can be attributed to a lack of consideration of indirect effects and additional stressors” (Schäffer et al., 2018, p. 28).

Liess et al. (2021) used the SPEAR indicator to evaluate the composition of the macroinvertebrate communities at the 124 stream sites they monitored in Germany. Comparing measured exposure and SPEAR values at the monitoring sites, they concluded that stressors “showing no or only minor associations with invertebrate-related endpoints include urban toxicants such as pharmaceuticals, heavy metals, and street run-off” (p. 4), and that pesticides are the dominant stressors. Neale et al. (2020), who assessed a subset of the monitoring sites investigated by Liess et al. (2021), reported that all “observed in vitro effects were dominated by street runoff chemicals such as 2-benzothiazolesulfonic acid” (a vulcanizing agent) and concluded that “Rain events clearly pose a threat to water quality in small streams, and analysis of pesticides alone cannot adequately judge the toxicological impact unless analytical monitoring is complemented by bioassays” (p. 8287).

A comparison of the conclusions of Liess et al. (2021) and Neale et al. (2020) from the same monitoring campaign raises the question of whether the effects on aquatic organisms described by the SPEAR index are primarily due to PPPs. To answer this question, a thorough evaluation of the effects of other chemical compound classes on the aquatic communities is needed. Furthermore, the evaluations presented by Liess et al. (2021) provide no insights regarding the impact of abiotic environmental parameters such as stream type, stream morphology, or catchment characteristics, which might have an influence on the SPEAR index (Rasmussen et al., 2016; Weiss et al., 2023). An in-depth analysis of these parameters would be needed to avoid “a lack of consideration of … additional stressors,” as mentioned by Schäffer et al. (2018, p. 28).

### Field-based effects

Schäffer et al. (2018) state that the examples they provide “reveal that the risk assessment requires a revision… Until such a revision process has been carried out, the safety margins (or: uncertainty factors) should be increased” (p. 34). These safety factors are used in combination with the hazard characterization of a PPP (i.e., determination of effect concentrations that describe which effect will occur to what extent at which exposure level) to derive maximum RACs in various environmental compartments. A PPP can only be authorized in the EU if the RAC is not exceeded by the predicted environmental concentration (PEC).

Liess et al. (2021) hypothesize that “synergistic increase of pesticide toxicity due to the presence of additional toxicants,” synergistically acting environmental stressors, and repeated insecticide exposure pulses “are the reason for the high field sensitivity of vulnerable species and the associated increased extrapolation factor identified” (p. 8). They claim that these “effect-determining factors and their related processes … are generally not considered in the aquatic risk assessment” and “suggest to calibrate the assessment factors applied in pesticide regulation integrating field-based findings” (p. 9). However, Neale et al. (2020) concluded from the same sampling campaign “that non-pesticide chemicals and even typical wastewater-derived chemicals were found at sites assumed to be largely free from wastewater effects prior to the study” (p. 8287). Looking at the density of small wastewater treatment plants at smaller brooks in rural areas (e.g., via the official ELWAS GIS tool [MUNV, n.d.]) their effluents may be an additional source of toxicants upstream of most sampling sites. Considering these aspects, it can be questioned whether applying higher assessment factors in pesticide regulation would substantially reduce the overall risk to aquatic communities. Increasing safety factors cannot reduce contaminations above the regulatory acceptable levels when they result from unauthorized application and handling or from spillages, accidents, or other unintended inputs (for example wastewater from point sources).

## Appropriateness of predictive exposure models

Citing Bonmatin et al. (2015) and Chiaia-Hernández et al. (2017), Schäffer et al. (2018) claim that “predictive models are in many cases erroneous” and that pesticides, e.g., neonicotinoids, “are detected in the soil for a longer period than was predicted in the studies used for authorization” (Schäffer et al., 2018, pp. 20–21).

However, these articles do not support this conclusion. Concentrations of imidacloprid detected in soil one year after application (Bonmatin et al., 2015) were within the expected range considering the dissipation half-life (DT50) of the active substance and therefore do not contradict the results from regulatory exposure predictions. Chiaia-Hernández et al. (2017) analyzed dried soil samples stored under cool conditions intended to prevent degradation. Detections of imidacloprid in these soil samples therefore do not provide evidence for high persistence under field conditions. Further large-scale soil monitoring studies involving multiple active substances (Silva et al., 2019) confirm the validity of soil exposure predictions.

Schäffer et al. (2018) also cite Vonberg et al. (2014), who reported that measured concentrations of active substances of PPPs applied on maize exceed the groundwater limit value of 0.1 µg/L, whereas the PECs for these compounds did not. The authors discuss the application of PPPs on agricultural fields according to good agricultural practice as cause for the observed exceedances, but do not consider the impact of additional sources from nonagricultural uses on the groundwater bodies investigated. Such uses include applications on railway lines and industrial areas kept free of vegetation by applying herbicides at much higher use rates than allowed on agricultural practice (see *Long-term detectability of PPP residues in groundwater* section).

Regarding surface water, Schäffer et al. (2018) cite articles such as Münze et al. (2017) and Knäbel et al. (2012) as proof of the inability of predictive models to reflect exposure in water bodies. However, Münze et al. (2017) report concentrations influenced by point sources (samples taken downstream of wastewater treatment plant [WWTP] effluents) and Knäbel et al. (2012) compiled concentrations from field studies where the impact of point and/or nonagricultural sources cannot be ruled out. Entries of active substances and/or metabolites into water bodies from these sources are not the result of PPP application on agricultural fields according to good agricultural practice and, therefore, these sources are not considered in the regulatory process of PPPs. Consequently, the reported concentrations are inappropriate for a comparison to PECs from the regulatory context. Inferring deficiencies of predictive models from such a comparison is not correct.

Also, Liess et al. (2021) consider the regulatory exposure models inappropriate for simulating exposure under field conditions and state that “monitoring-based findings show that … measured environmental concentration (MEC) was higher than the predicted environmental concentrations” (p. 5). They infer this statement from analyzing chemical monitoring data collected in 124 stream sites in Germany for 75 active substances and focusing on those 20 substances exceeding the current substance RAC most frequently.

Liess et al. (2021) and Halbach et al. (2021) showed that for 15 of these 20 substances, RAC exceedances were observed in less than 6% of the samples (terbuthylazin, nicosulfuron, lenacil, diflufenican, thiamethoxam, S-metolachlor, foramsulfuron, dimethenamid-p, acetamiprid, pirimicarb, mesotrione, dimoxystrobin, bromoxynil, MCPA, and azoxystrobin). For the substances imidacloprid, methiocarb, fipronil, and chlothianidin, RACs were exceeded in 8.4%, 9.3%, 12.5%, and 14.3% of the samples, and RAC exceedances were observed most frequently for thiacloprid (34.4%) (Halbach et al., 2021). Observed RAC exceedances for thiacloprid, clothianidin, imidacloprid, terbuthylazine, lenacil, and nicosulfuron can be attributed to the fact that the RAC values used by Liess et al. (2021) and Halbach et al. (2021) were lower than the RAC values set in the authorization process and for deriving currently valid risk mitigation measures. Comparing the measured concentrations of these six substances reported by Liess et al. (2021) with the RAC values set in the authorization process, RAC exceedances of more than 6% occur for clothianidin, fipronil, and methiocarb (the latter two are also used as biocides) (see online supplementary material chapter 5).

Liess et al. (2021) also provided PEC values for 16 of the 20 active substances. For three out of 16 substances, the PEC was always higher than the MEC, whereas for the remaining 13 compounds, the maximum MEC was higher than the PEC. However, the MEC exceeded the PEC in less than 6% of the samples and these exceedance rates may include entries from point and/or nonagricultural sources, as mentioned before. Therefore, it can be concluded that the exposure predictions are protective of greater than 90% of the monitored exposure situations in space and time. These results demonstrate that the exposure predicted for the 20 compounds is conservative in the vast majority of cases.

In summary, a dedicated analysis of the examples presented by Schäffer et al. (2018) and the evaluation presented by Liess et al. (2021) and Halbach et al. (2021) demonstrate that a systematic underestimation of real environmental concentrations is not the case.

However, it cannot be ruled out that there is a need for improvement. If model weaknesses are identified, they are addressed—as shown by the example of Bach and Hollis (2012), who identified the short simulation period of one year as a weakness of the FOCUS surface water approach (FOCUS, 2001). The FOCUS work groups of the FOrum for Co-ordination of pesticide fate models and their Use (FOCUS DG SANTE—ESDAC—EC, n.d.) developed guidance for the use of simulation models in the EU regulatory environment and published reports and initial guidance documents for PEC calculations at EU level in groundwater (FOCUS, 2000), surface water (FOCUS, 2001), and degradation kinetics (FOCUS, 2006). Model weaknesses and version control issues were addressed by FOCUS groups and later transferred to ESDAC and EFSA. Addressing the concerns about the limited simulation period, the EFSA working group “FOCUS surface water repair” has been formed to implement multiyear runs to reflect the influence of 20 meteorological years on the predicted surface water concentrations (Adriaanse et al., 2020a, 2020b).

## Surface water exposure

### Elements of the regulatory exposure assessment

Schäffer et al. (2018) mention that Knäbel et al. (2012) showed “that the standard models for prospective exposure assessment (so-called FOCUS scenarios) used in the EU needed to be replaced by models that factor in regional features such as location-specific climate data” (p. 21).

In this general statement, Schäffer et al. (2018) misperceive the basic principles of regulatory modeling, i.e., a validated mechanistic exposure model is applied to representative location-specific scenarios. This basic principle is also implemented in the FOCUS approach to predicting surface water exposure for regulatory risk assessment. According to EU Reg 1107/2009, environmental concentrations in surface waters are predicted following the recommendations of FOCUS that are mandatory for the authorization process (FOCUS DG SANTE—ESDAC—EC, n.d.). Exposure is simulated for a set of FOCUS environmental scenarios using the FOCUS surface water models (FOCUS, 2001). Each FOCUS scenario reflects a small water body in a landscape described by realistic worst-case assumptions on pedoclimatic conditions that are representative of important agricultural areas in Europe. The FOCUS exposure models are validated mechanistic models implementing the transformation and transport processes that determine the behavior of substances in the environment (Adriaanse et al., 2020a, 2020b; Chen et al., 2017; Singh & Jones, 2002; Zhang & Goh, 2015). Model input parameters describing the environmental fate of the active substance and metabolites (e.g., degradation, sorption) are selected according to the EFSA conclusion on the active substance, which summarizes the results of the European member state peer-review process (EFSA, n.d.-b).

Environmental concentrations are predicted by considering details of the intended PPP application on agricultural fields (e.g., application type, timing, applied amount) compliant with good agricultural practice (GAP) (CEC, 1999; FAO, 2003). Fields treated according to GAP can be referred to as “diffuse agricultural sources” in the regulatory context, as it is assumed that transport from these fields to adjacent waterbodies may occur via spray drift, runoff, and/or drainage. The regulatory exposure assessment considers neither point sources as a result of accidents or noncompliant handling of PPPs (i.e., sources such as effluents from farmyards, machinery wash bays, or effluents from WWTPs into brooks) nor nonagricultural sources (e.g., biocides, industrial uses). These sources are regulated by other pieces of legislation, e.g., the German National Wastewater Regulation (BMJ, 2022) implements the specifications of the Water Framework Directive (EPC, 2000) into national law.

### Comparison of regulatory exposure predictions and measured concentrations

Schäffer et al. (2018) state that “Pesticide residues can also be found in water in higher concentrations than were predicted in the exposure assessments” (p. 21).

There can be various reasons for such exceedances, some of which will be discussed later in this publication. First, it needs to be noted that a meaningful comparison of measured and predicted exposure to determine the appropriateness of predictive models is only possible with sufficiently resolved application data in space and time from monitored water catchments. Without this information, it is not possible to thoroughly elucidate sources and entry pathways relevant for the observed exposure (Schönenberger et al., 2022).

Schäffer et al. (2018) cite Knäbel et al. (2012) as proof for the inability of predictive models to reflect exposure in water bodies. Knäbel et al. (2012) compared measured environmental concentrations in surface water to environmental concentrations that were predicted using the FOCUS models and scenarios. They stated that, in a considerable number of cases, MECs of insecticides extracted from 22 field studies were underestimated. Bach and Hollis (2012) reviewed the 22 studies and found that only three of them (with 17 measured field concentrations for two insecticides) are appropriate for a comparison with FOCUS predictions, because (i) they represent agricultural practice and pedoclimatic conditions for which the EU FOCUS scenarios were selected and (ii) exposure was caused by runoff, drainage, and spray drift. The other studies were conducted outside the EU and/or diffuse agricultural pollution was not the only source of exposure, as farmyard drainage effluents, irrigation return water, wash-off from roads along the fields, or structural pest control (i.e., building and property pest management) were mentioned as additional sources. Predicted environmental concentrations from regulatory models reflect the impact of diffuse agricultural sources for a defined set of agri-environmental scenarios. Measured environmental concentrations from monitoring programs are influenced by additional sources of pollution and different environmental conditions. Therefore, a comparison of PECs and such MECs is not suitable for assessing the appropriateness of regulatory models.

Even if MEC and PEC reflect similar environmental conditions and applications were done according to good agricultural practice, single temporal and spatial exceedances of PECs by MECs are expected. This is due to PECs in surface water calculated according to FOCUS being considered as representing an overall 90^th^ percentile aquatic exposure and, therefore, to be protective of 90% of all exposure situations in the EU in space and time (EFSA PPR Panel, 2013). With regard to this agreed exposure assessment goal, occasional PEC exceedances do not indicate that “the FOCUS modeling approach is … not capable of predicting the actual field exposure levels” as postulated by Knäbel et al. (2012, p. 8403). Recently, the protectiveness of the FOCUS surface water approach was exemplarily shown by comparing PEC in surface water with measured concentrations from a long-term, high-frequency surface water monitoring (Hörold-Willkomm et al., 2024).

In conclusion, it can be stated that a comparison of monitoring and modeling needs to be done with more accurate comparisons and an appropriate level of detail to judge whether a measured concentration really originates from application according to GAP.

### Role of point sources

Schäffer et al. (2018) consider MECs exceeding PEC as “a result of surface runoff and wastewater treatment plants, although this should have been ruled out in the authorization” (p. 21). Inputs from WWTPs are not considered in the risk assessment of PPPs. Therefore, the occurrence of ecotoxicologically relevant pesticide concentrations downstream of WWTP effluents is not evidence of shortcomings of the authorization process.

Schäffer et al. (2018) cite Münze et al. (2017), who investigated pesticide concentrations upstream and downstream of WWTP outlets and concluded that pesticides from wastewater treatment plant effluents affect invertebrate communities. This type of pesticide entry into water bodies is considered as a point source of pesticide exposure as mentioned before and is not considered in the regulatory environmental exposure assessments. Therefore, comparing MECs affected by point sources with PECs only considering diffuse sources is not appropriate for inferring deficiencies of predictive models. Consequently, the investigation by Münze et al. (2017) is not proof of deficiencies in the regulatory system.

The relevance of point sources for the entry of PPPs into surface waters in Germany was previously described in Bach et al. (2000, 2001, 2005) for some important river catchments. Bach et al. (2005) estimated the contribution/fraction of entries from nondiffuse sources (e.g., from WWTPs) into surface water at 65% to 95% of the total pesticide river load.

Similarly, Korkaric et al. (2023) estimated nondiffuse sources to be around 62% (40% farmyards, 22% hydrological shortcuts) and the contribution of diffuse sources to be around 38% (22% runoff, 11% drainage, and 5% drift).

Gerecke et al. (2002) investigated the origin of pesticide concentrations in the effluents of WWTPs in Switzerland and demonstrated the relevance of elevated pesticide concentrations in these effluents for measured concentrations in receiving water bodies. Buerge et al. (2009) demonstrated that acesulfame was an “ideal marker” for contamination of groundwater by wastewater. Kahle et al. (2009) came to a similar conclusion for carbamazepine as a “promising marker” for wastewater entries into ground and surface water. Schmidt et al. (2005) elucidated the causes for 151 reports of findings. The main causes of findings in groundwater were infiltration of surface water contaminated, e.g., by runoff or drainage (25%), wastewater discharges (17%), point sources (11%), and poor-quality monitoring wells (10%). The contribution of effluents from small scale WWTPs to the pollution of water bodies was demonstrated by Karfusehr et al. (2018), who analyzed groundwater samples for acesulfame and carbamazepine and found these indicators of human wastewater downstream of farms in pore-water aquifers. Hillebrand et al. (2012) showed this for caffeine, which was discharged with leaking wastewater into a karst aquifer.

For 11 of their 124 monitoring sites in small agricultural streams, Liess et al. (2021) reported sources of nonagricultural pollution, such as small WWTPs. Neale et al. (2020), who investigated 44 of the 124 sites, actually identified 32 sites as “impacted” or “likely impacted” by wastewater effluents, based on the detection of the wastewater markers acesulfame, sucralose, carbamazepine, cyclamate, saccharin and caffeine (see online supplementary material chapter 4). Moreover, 50% or more of the samples analyzed by Liess et al. (2021) showed at least one of the wastewater markers acesulfame, caffeine, and/or the pharmaceuticals carbamazepine and oxypurinol, indicating domestic discharges into the monitored streams. The numerous wastewater markers found at their monitoring sites clearly show the impact of effluents from settlements and farmyards in the samples. This underlines the need for more effective treatment of wastewater from sewage treatment plants (e.g., by installing a fourth treatment stage), which should be included based on the relevant wastewater legislation.

Despite the known relevance of point sources and the demonstrated impact of wastewater on several of the monitored sites, Liess et al. (2021) predominantly attributed the PPP concentrations measured to product application in line with good agricultural practice on upstream fields. In consequence, they conclude that the regulatory system underestimates PPP concentrations in surface waters and therefore needs adjustment. However, conclusions about the effectiveness of the regulatory system should only be drawn based on samples taken at sites upstream of effluents from settlements and farmyards.

### Relevance of application data and information on risk mitigation measures

Liess et al. (2021) hypothesize that the PEC exceedances they reported were “possibly either due to unauthorized application rates, faulty exposure modelling, failure to consider multiple applications in the river basin, or overestimation of the predicted effectiveness of risk reduction measures” (p. 6). However, data supporting these hypotheses are not presented.

Application data (plot resolution, daily scale of uses) for the catchments of the monitored streams in the investigated period were not considered. Therefore, clarification of actual application rates and occurrence of multiple applications is impossible. Elucidation of potential errors in modeling is not possible because no details were presented on either the PEC calculations or the regulatory relevant risk mitigation measures considered for modeling (e.g., spray drift or runoff reduction).

Moreover, no information was provided on risk mitigation measures actually used at the monitored stream sites (e.g., use of drift reducing technology during application or presence of vegetated filter strips to mitigate runoff). At the same time, photographs from the sampling locations presented in Müller and Hitzfeld (2020) show that some fields adjacent to sampled stream sites are located on slopes directly at the break-off edge to the stream, i.e., without any vegetated filter strips implemented. Therefore, Liess et al. (2021) lacked a sound data basis for inferring that the effectiveness of regulatory relevant risk mitigation measures is overpredicted.

Hypothesizing on the effectiveness of measures to mitigate risks from diffuse agricultural sources, Liess et al. (2021) do not comment on other exposure pathways for PPPs, although the detection of numerous wastewater markers at the monitored sites showed the impact of effluents from settlements and farmyards (Neale et al., 2020; see also online supplementary material chapter 5). Vormeier et al. (2023) evaluated the data presented by Liess et al. (2021) and calculated widths of vegetated buffer strips including these point sources (“…these protective VBS widths include all point source inputs…” [p. 6]). However, mitigating inputs from point sources such as WWTPs and accidental spillages via vegetated buffer strips is not possible (see *Role of point sources* section).

Discharges from treated areas can also occur via the farm road network into gullies (and then into surface waters) (Schönenberger & Stamm, 2021). High special crops on steep slopes, such as vineyards, are particularly susceptible to this, as shown by Bereswill et al. (2012) and (2014), who suggest, among other things, the greening of vineyards and the implementation of grassy buffer strips to field paths/dirt roads as a remedy.

## Soil exposure

### Elements of the regulatory exposure assessment and PPP residues in soil

Schäffer et al. (2018) state “substances remain in the soil for decades, although significantly shorter retention times …were anticipated in the authorization documents” (p. 21). With their statement, the authors are presumably referring to the degradation times provided in the soil exposure assessments required according to EU Reg 1107/2009. They report that “numerous pesticides have also been detected in Portuguese, Spanish, and Finnish soils long after their usage” (p. 21). This observation is not surprising, because after the period of one half-life has passed, 50% of the amount originally applied is still present in soil. Furthermore, many substances follow biphasic degradation kinetics, with a fast initial degradation and a slower second phase. Using the DT50 from the initial phase for extrapolating beyond the fast phase leads to an underestimation of the residual concentrations in soil. Overall, care must be taken that long-term predictions of residues are not oversimplified by assuming single first order kinetics in all cases.

Soil exposure assessments are carried out with model calculations based on degradation rate parameters derived from either experimental laboratory studies or field studies and consider the potential long-term accumulation behavior under realistic worst-case situations. Authorizations are only granted if the resulting PECs are sufficiently lower than the endpoints from ecotoxicological studies on soil organisms to ensure no unacceptable effects occur. The mere detection of a PPP in soil, even over longer periods, is not surprising for many compounds, given their published time required to degrade 50% of the initial concentration (DegT_50_) values. A prominent example is the persistence of the fungicide copper used in permanent crops (Burandt et al., 2024; Kuhne et al., 2017) and the effects on soil organisms (Riepert, 2009; Zubrod et al., 2015). Also, the mere detection of a substance in soil (or water) is not necessarily a reason for concern. Instead, measured concentrations should be compared with RACs in EFSA endpoint lists to assess whether there is reason for concern. The models also consider that degradation is dependent on the respective kinetics of the substance as well as the prevailing environmental conditions, such as moisture and temperature. Exact model definitions and examples have been published by regulatory authorities (e.g., EC, 2021; FOCUS, 2006; NAFTA Technical Working Group on Pesticides, 2022; various renewal assessment reports (RARs) and list of endpoints on the EFSA website (EFSA), n.d.-b).

Schäffer et al. (2018) state that “to date, there are only a few examples of studies on the degradation under realistic conditions…” (p. 24). This statement is not correct, because field studies for PPP are often triggered during the regulatory process. The DegT_50_ or DegT_90_ (time required to degrade 90% of the initial concentration) is determined in laboratory studies. If 60 days (DegT_50_) or 200 days (DegT_90_) are exceeded (EC, 2013a, 2013b), at least four outdoor terrestrial field dissipation studies under real-world conditions are mandatory, typically conducted over two years across Europe. A synopsis of data from several terrestrial field dissipation studies across Europe is compiled in Sur (2014). Furthermore, examples for accumulation studies are provided in online supplementary material chapter 2, where two long-term soil monitoring studies are compared to model predictions.

### Comparison of regulatory exposure predictions and measured concentrations

Schäffer et al. (2018) cited Bonmatin et al. (2015), who were concerned about the detection of imidacloprid in soil “in 91% of the samples (>0.1 µg/kg), although only 15% of the sites had been planted with treated seeds during the same year” (p. 40). Using degradation rates and exposure modeling helps put their findings into context. Calculations based on the ESCAPE regulatory model (Fraunhofer IME, 2008) show that residues of imidacloprid can still be detected > 0.1 µg/kg several years after the last application (> 4 years). These concentrations have passed the regulatory risk assessment, i.e., are not expected to cause unacceptable ecotoxicological effects. In conclusion, the findings by Bonmatin et al. (2015) do not contradict the results from regulatory exposure predictions for seed dressings in France and validate the regulatory calculation mentioned above rather than contradicting it.

Schäffer et al. (2018) cite Chiaia-Hernández et al. (2017), who reanalyzed soil samples taken by the Swiss soil monitoring program Nabo from 1995 to 2008. These samples were stored in plastic containers under cold and dry conditions in a soil archive for more than nine years (Hämmann & Desaules, 2003). The procedure described leaves the samples in conditions that slow down or even stop microbial degradation processes (OECD, 2016). Consequently, it is no surprise that the authors could still find residues of the pesticides applied during the sampling period in the archived samples. It was also not reported by Chiaia-Hernández et al. (2017) that they detected PPPs “in soil samples taken now,” as wrongly stated by Schäffer et al. (2018). Therefore, the study by Chiaia-Hernández et al. (2017) does not support the claims on soil persistency made by Schäffer et al. (2018).

More recently, an extensive comparison of soil exposure monitoring data with PECs used for the EU approval of active substances has been carried out by Silva et al. (2019). The authors analyzed 317 topsoil samples across the EU for 76 pesticides and only found slight occasional exceedances of the regulatory predictions for not more than three compounds, which can be attributed to higher use rates registered in some of the member states than the rates assessed by EFSA in the so-called “representative use.” Nevertheless, these higher use rates still passed the risk assessment in the member states. These very large monitoring studies demonstrate the validity of soil exposure predictions performed during the approval process and the protectivity of the regulatory system.

## Groundwater exposure

### Regulatory exposure assessment

Schäffer et al. (2018) state that “the active substance and degradation products of the herbicide atrazine can still be detected in groundwater, approximately 25 years after the ban on the herbicide in Germany” although “several hundred days was estimated as the maximum time for the active substance to be metabolized to half of its original amount (the half-life)” (pp. 23–24). The authors consider these detections to be another proof for their claim that “predictions based exclusively on models are not reliable” and that “the evaluation models underlying such predictions must undergo a validation more strongly informed by real-world conditions than has been the case until now” (p. 23).

For the groundwater exposure assessments required by EU Reg 1107/2009, PECs in groundwater are calculated using FOCUS groundwater models and scenarios (EC, 2014; FOCUS, 2000, 2009). The predictions were validated using outdoor lysimeter experiments and field leaching experiments that allow for the investigation of the leaching behavior of compounds under real-world conditions (e.g., Hardy et al., 2008; Jene et al., 1999; Klein et al., 1997, 2000, 2019; Sur et al., 2022; Vanclooster et al., 2000).

When simulation models were introduced into the leaching assessment in the early 1990s, the approval for atrazine was not extended. Atrazine was also used outside of agriculture with high application rates on permeable substrates, such as railway ballast and in industrial facilities. The implications of this particular use are presented in the *Long-term detectability of PPP residues in groundwater* section and the online supplementary material chapter 6, for the study by Vonberg et al. (2014) as referenced by Schäffer et al. (2018). In conclusion, atrazine is a special example where first the high application rates on permeable, nonagricultural substrates and then the use of the substance itself were withdrawn. Therefore, the example should not be used to question the extensive validations with other active substances for uses on arable land. Generally, it cannot be ruled out that compounds are detected several years after application. This is not in itself a concern when regulatory thresholds are not exceeded. The reasons for this are explained in more detail in the *Prediction of long-term leaching in groundwater by modeling* section in the online supplementary material chapter 3.

### Long-term detectability of PPP residues in groundwater

To support their claim about the deficiencies of regulatory exposure modeling, Schäffer et al. (2018) cite the monitoring studies of Vonberg et al. (2014), who investigated the atrazine concentrations in an aquifer with intensive agricultural land use but also railway tracks, urban areas, and industrial areas in the catchment. Vonberg et al. (2014) reported that the “monitoring data shows that even 20 years after the ban of atrazine, the groundwater concentrations of sampled OW [observation wells] remain on a level close to the threshold value of 0.1 μg/L without any considerable decrease” (p. 294).

Vonberg et al. (2014) attribute the atrazine findings in groundwater exclusively to the agricultural use of atrazine in maize. The authors did not consider that the railway facilities, parts of commercial and industrial areas, sports facilities, and urban areas in the catchment were also subject to weed control measures using herbicides (e.g., on paths, squares, industrial facilities). Therefore, the authors’ inferences regarding the exposure assessment are based on incomplete assumptions on application areas, dosages, and total amounts applied in the area. Such omissions and inconsistencies make it impossible to draw valid conclusions about substance behavior and do not justify demands for a revision of the current authorization procedure.

The authors present their results as evidence of deficiencies in the current authorization procedure. However, with the help of a landscape analysis of the study area, which takes into account typical application rates of atrazine in agricultural and nonagricultural applications, it can be shown that the active ingredient quantities that could be applied on the railway lines and a centrally located industrial plant in the study area alone correspond to about half of the quantity that could be applied to maize (for details on this GIS analysis, see online supplementary material chapter 6). The application rate was also several times higher on railway lines with permeable ballast beds than on arable land. Finding herbicides used almost exclusively in the railway track area (BT, 1989) may provide evidence of nonagricultural applications in the catchment area. The leaching potential from treated railway tracks and transport with the groundwater flow over long distances to below agricultural land has been intensively studied (LU, 2002; LUBW, 2001, 2024). It was only long after the approval of the high application rates on track and noncrop areas, which contributed to the exceedances of the threshold value of 0.1 μg/L, that this threshold value was put into effect. Most of these high-rate applications were prohibited in the late 1980s after introduction of the new threshold.

### Official reports on groundwater and drinking water quality

The “Reports on Groundwater Quality—Plant Protection Products,” regularly edited by the German Working Group on water issues of the Federal States and the Federal Government (LAWA, 2019), provide information on the findings of PPPs in groundwater in Germany. In 2019, the report evaluated the state groundwater monitoring data available from 1990 to 2016. The result “…clearly shows that the overall situation of groundwater contamination by pesticides has improved considerably over time. The proportion of monitoring sites where the threshold value of the Groundwater Ordinance of 0.1 μg/L is exceeded continuously decreased from 9.7% in the period 1990 to 1995 to 3.8% in the current period 2013 to 2016” (LAWA, 2019). This also holds true for atrazine: “The time trend evaluation … based on consistent monitoring sites over the last four observation periods (2001 to 2016) shows a distinct decline in detections in groundwater.”

The German Federal Ministry of Health and the Federal Environment Agency report on drinking water quality states the concentrations of almost all monitored and reported active substances and their metabolites were below the limit value of 0.0001 mg/L in the reporting period (UBA & BMG, 2021). The concentrations of active substances and metabolites in raw water abstracted for drinking water production can be found in the reports on the corresponding website of the German Centre for Water Technology (TZW) (e.g., Castell-Exner et al., 2023). Obviously, and contrary to the claims by Schäffer et al. (2018), the regulatory system was working quite effectively over recent years to reduce concentrations of active substances reaching water bodies and to protect drinking water.

## PPP approval procedure

### Stepwise authorization after assessing results from monitoring

Schäffer et al. (2018) suggest a “tiered authorization procedure” with a provisional approval for a limited use of new substances in selected areas, accompanied by untargeted chemical and/or biological monitoring in these areas over a longer period. They see the benefit of monitoring in “the continuous gathering of knowledge about the fundamental suitability of current exposure models and concepts for meeting the aspired conservation objectives” (p. 35).

Untargeted monitoring in limited areas involves uncertainties. Pedoclimatic or ecological conditions within one area might represent a worst-case scenario for one environmental situation but can represent an average or even a best case for another. For instance, areas that represent a worst-case situation regarding off-site transport (e.g., due to leaching into groundwater or runoff to surface water) cannot represent a worst-case scenario regarding onsite accumulation in soil.

Data from untargeted monitoring are not appropriate for validation/calibration of predictive models considered in the authorization procedure unless it can be clarified that observed exposure or effects can be attributed to the application on agricultural fields in line with good agricultural practice (see *Appropriateness of predictive models* section).

The concept of investigations in selected areas is already included in the current tiered authorization procedure. If critical areas are identified in a lower-tier assessment, targeted higher-tier field testing of new active substances can be required to investigate, e.g., degradation (terrestrial field dissipation studies or accumulation studies), leaching to groundwater (outdoor leaching studies), or effects in aquatic and terrestrial environments. Targeted field testing needs to be conducted with the highest predicted product use rates and under worst-case environmental conditions. New modeling concepts such as spatially distributed approaches can help identify these conditions (ESDAC, 2019). Overall, such targeted approaches provide more precise insights into critical parts of the risk assessment than an untargeted monitoring program (Gimsing et al., 2019).

### Effect monitoring

Schäffer et al. (2018) suggest untargeted monitoring should be conducted over a longer period to confirm the absence of adverse effects. In the absence of adverse effects, the use area could gradually be extended until authorization for long-term use over larger areas is granted.

The ambition of linking observed effects on biota to chemical exposure based on untargeted monitoring is hampered by the unavoidable influence of confounding factors/stressors. Discerning the influence of factors such as weather, food availability, predation, or competition is much more challenging in landscape-based monitoring than it is in a dedicated field study with statistical replication and untreated control plots. This will be aggravated if the investigation aims to study potential effects under different ecological boundaries or over several years, e.g., to cover long-term effects, different meteorological conditions, or landscape variability.

Dittrich et al. (2019) investigated bird communities in chlorpyrifos-treated and untreated (organic) citrus and apple orchards in Spain and the United Kingdom. Similarly, in a huge field study, Heimbach et al. (2016), Sterk et al. (2016) and Osterman et al. (2019) investigated the effects of clothianidin seed-treated winter oilseed rape on three species of pollinating insects (*Apis mellifera*, *Bombus terrestris*, and *Osmia bicornis*). Details on these studies are given below.

These examples of effect-monitoring studies show that risks that were not excluded at lower tiers of the assessment did not materialize under real-world conditions. However, there was speculation that confounding factors such as differences between sites and a lack of (statistical) power masked small, undesired effects in the Heimbach et al. (2016) study (Bailey & Greenwood, 2018). They highlight the difficulty of using monitoring studies to conclusively show absence of effects in the field.

### Monitoring in the current regulatory framework

Schäffer et al. (2018) suggest that “there is a need for a suitable measuring system (monitoring) in the post-authorization phase” (p. 35). However, the regulatory framework already provides the option for authorities to require monitoring studies for approval during the authorization process, including for new substances (Aden et al., 2002). Such studies are described and evaluated in detail, e.g., in EFSA conclusions or in RARs. Additionally, regulatory authorities may consider existing monitoring programs from third parties for authorization decisions.

Recent examples of environmental monitoring data considered in the approval process are available for the areas of exposure and effect monitoring.

- Targeted groundwater monitoring studies were requested by authorities to investigate the occurrence of the 1-2-4-H-triazole metabolite of triazole fungicides in areas of intensive use of triazole containing PPPs (BVL, 2020, 2022). After reviewing the monitoring data (see online supplementary material chapter 7), the authority concluded that their initial concerns were adequately addressed and resolved.
- The effectiveness of vegetated buffer strips to protect adjacent streams from terbuthylazine runoff was closely investigated with automatic samplers at four sites over four years (Bischoff et al., 2003; EFSA, 2011; EFSA et al., 2017; see online supplementary material chapter 8).
- Dittrich et al. (2019) investigated bird communities in chlorpyrifos-treated and untreated (organic) citrus and apple orchards in Spain and in the United Kingdom, respectively, over a total of six years. They found that insectivorous species were the most abundant feeding guild in both orchard systems, despite the regular applications of chlorpyrifos over many years. Furthermore, none of the species declined in abundance after consecutive years of insecticide use. Scarcity of nesting sites might have had greater influence than the application of chlorpyrifos, because a population of two insectivorous tit species was successfully established inside the apple orchards following the installation of nest boxes.
- Similarly, in a huge field study, Heimbach et al. (2016), Sterk et al. (2016), and Osterman et al. (2019) investigated the effects of clothianidin seed-treated winter oilseed rape on three species of pollinating insects (*Apis mellifera*, *Bombus terrestris,* and *Osmia bicornis*). They found no detrimental effects on any of these species.

## What do we propose?

### Looking beyond PPP authorization

The major challenges for agriculture in the twenty-first century will be addressing climate change, managing natural resources such as soil and water more sustainably, and promoting biodiversity, while feeding a rapidly growing world population. In resolving this conflict of goals, the sustainable use of PPPs plays an important role, along with others. However, it will not be possible to resolve these conflicting goals simply by “tightening-up” risk assessment alone, thus achieving the improvements for soil, water, and biodiversity stated by Schäffer et al. (2018), as detrimental effects stem from sources other than proper and intended use according to the label instructions for PPPs (as outlined in the previous sections).

Although we agree that the risk assessment system should be continuously evolving, in our view, the most efficient approach to resolving this conflict of goals is enhancing landscape diversity; mitigating or even eliminating point sources; and maintaining an adequately complex, efficient, and flexible authorization procedure.

### Biodiversity

The input of PPP on fields can and should be reduced as far as possible by consequent application of integrated pest management practices and precision application. We also support the consistent protection of marginal habitats, which should not be used for food production and should be maintained without chemical inputs. The same should apply for riparian buffer strips of sufficient size (which will depend on local conditions). The protection and creation of interconnected hedges, small woods, riparian strips, ponds, and uncropped strips or lark windows in very large fields will significantly contribute to improved biodiversity within the agricultural landscape. It is essential to choose measures that fit with regional landscape conditions (Hertzog et al., 2023). Cooperation between farmers, government, and regional nature conservation experts is a very promising way forward, as can be seen in the Netherlands (Terwan et al, 2016; Westerink, Jongeneel, et al., 2017; Westerink, Opdam, et al., 2017; see also online supplementary material chapter 9).

### Point sources

We support continuous training of farmers to raise awareness of the relevance of compliant handling of PPPs. This will help reduce the impact of agricultural point sources, which were recognized decades ago as a major contributor to PPP pollution of water bodies (Bach et al., 2000, 2005) but which, despite great improvements, still remain an important issue. A recent example again shows how training farmers in good agricultural practice and stewardship measures can successfully reduce environmental exposure (Schuster et al., 2023). Connecting more small settlements (including farms) to central wastewater treatment plants and installing additional treatment steps would further reduce the impact nonagricultural chemicals (e.g., biocides, industrial and urban wastewater, and road runoff) discharged into streams have on biota.

### Authorization procedure

We generally support advancing the regulatory system whenever needed. A more efficient authorization procedure would speed up innovation and help to bring safer products to the market faster. A multi-stakeholder workshop, “Plant Protection Products Authorization 2030,” organized by the German regulatory authority BVL, identified options for improving efficiency and clarity in the area of developing a common understanding of guidance documents and interpretation of the current scientific and technical knowledge, thus enabling a harmonized and predictable application of the EU authorization regulation (BVL, 2024; Cramer, 2022).

Repeated calls to tighten the authorization procedure and also generally reduce the number of PPPs do not promote biodiversity per se. On the contrary, via the resulting required increase in food imports, they lead to the agricultural intensification of more land and possibly more sensitive landscapes outside Germany or other European countries. The discussion should rather focus on targeted risk mitigation measures that already facilitate a reduction in off-target transport of PPPs. For mitigation to become broadly effective, it must become part of the authorization process and part of product label phrasing. Certified digital advisory tools that determine the performance of local risk reduction measures would be the next step in improving and guiding farmers’ application choices. We therefore support the implementation of flexible toolboxes of mitigation measures, such as the Magpie toolbox (Alix et al., 2017), the more recent EU compendium (EC, 2024), and similar approaches available in the United Kingdom (LERAP), Sweden (Helper system), Switzerland (point system), and Italy (percentage system). Examples are in-row (band) applications, spot applications, conservation tillage, mulch seeding, micro-dams in maize and potatoes, cover crops, undersown crops in maize, greening of vineyards, and buffer strips at field margins close to farm tracks. The elements in these toolboxes result from concerns and suggestions by, e.g., Bereswill et al. (2012) and Bereswill et al. (2014).

## Conclusion

This article demonstrates that claims by various authors regarding the inadequacy of the authorization process for PPPs in Europe are not supported by scientific evidence. Concentrations of concern reported for pesticides or their metabolites in environmental media do not show the inadequacy of the authorization process when they are observed in environmental situations for which the EU regulatory scheme was not developed (e.g., PPP applications outside EU agricultural landscapes, PPP exposure due to agricultural point sources, or nonagricultural sources). Connecting more small settlements including farms to central wastewater treatment plants and installing additional treatment steps would further reduce the impact of agricultural point sources. In these cases, increasing the conservativeness of the EU authorization procedure would not lead to any improvement of the reported environmental situation. The authorization procedure in the EU is evolving continuously, and scientific findings make their way into the environmental assessment process. Observed environmental concentrations in the EU exceeding RAC values have been reported for only a few environmental situations and compounds. This suggests that, overall, the European regulatory system for PPP is protective. Further reduction of PPP exposure and risk from diffuse agricultural sources can be achieved by establishing the regulatory acceptance of a flexible management toolbox of mitigation measures. Digital decision systems can help identify the most effective combination of treatment and mitigation for individual fields.

## Supplementary Material

vjae007_Supplementary_Data

## Data Availability

The data can be accessed through the references provided.
