# Supplementary material for: The European regulatory system for plant protection products—cause of a “Silent Spring” or highly advanced and protective?
Source: Integr Environ Assess Manag. 2025 Jan 6;21(1):3–19. doi: 10.1093/inteam/vjae007 (PMC11804878; doi:10.1093/inteam/vjae007)
Supplement: vjae007_Supplementary_Data [file vjae007_supplementary_data.pdf]

**SUPPORTING INFORMATION TO**

# The European regulatory system for plant protection products – cause of a "Silent Spring" or highly advanced and protective?

Carola Schriever[1], Bernhard Jene[1], Herbert Resseler[2], Robert Spatz[3], Robin Sur[4], Arnd Weyers[4], Mark Winter[5]

[1] BASF SE, Agricultural Solutions, Environmental Fate, Speyerer Str. 2, 67117 Limburgerhof, Germany

[2] Syngenta Agro GmbH, Lindleystr. 8D, 60314 Frankfurt am Main, Germany (Retired)

[3] Syngenta Agro GmbH, Lindleystr. 8D, 60314 Frankfurt am Main, Germany

[4] Bayer AG, Crop Science, Environmental Safety, Alfred-Nobel-Str. 50, 40789 Monheim am Rhein, Germany

[5] Industrieverband Agrar e.V. (IVA), Wissenschaft und Innovation, Mainzer Landstr. 55, 60329 Frankfurt am Main, Germany

## CONTENTS OF THE SUPPORTING INFORMATION


1. Yields in organic and conventional crop rotations and self-sufficiency rates ............... 3
2. Methods of the prediction of long-term behavior in soil ............................................ 11
3. Predicting long-term behavior in groundwater by modelling ..................................... 15
4. Information on markers of wastewater effluents in small surface water bodies .......... 21
5. Exceedances of regulatory acceptable concentrations of plant protection products in small streams in agricultural areas in Germany ........................................................... 25
6. Contribution of non-agricultural uses to long-term detectability of PPP residues in groundwater ........................................................................................................ 34
7. Monitoring for regulatory purposes in groundwater .................................................... 40
8. Monitoring for regulatory purposes in surface water after runoff events .................... 43
9. Targeted promotion of biodiversity ............................................................................ 46

## 1. Yields in organic and conventional crop rotations and self-sufficiency rates

The purpose of this Supporting Information is to provide background information on the yield differences between organic and conventional farming to support reliable estimates of self-sufficiency rates for crops that are important for nutrition and food security.

### *Introduction*

Organic farming appears to be a simpler way of using fewer chemicals and supporting biodiversity. However, switching from conventional to organic production requires additional land resources to produce food, as organic farming produces less food per area than conventional farming. The greater the yield difference and the lower the level of self-sufficiency, the more additional resources and biodiversity would be consumed from elsewhere. Yield comparisons (organic vs. conventional) can be based on plot experiments, average yields per hectare (as reported in official harvest statistics) and at the level of full crop rotations typical of the farm management system. The literature indicates that comparisons intended to predict levels of self-sufficiency often fail to compare the whole production system considering the full rotation cycle. This Supporting Information shows some examples to illustrate that reliable calculations of the level of self-sufficiency and the level of imports required after conversion should be based on data representing the overall productivity level of the typical crop rotation of the farm management system.

### *Methodology*

Data were collected from published literature and market information from statistical offices on yields, yield comparisons and self-sufficiency. Note: Not all references refer to the same crop year and crop/product groupings may differ slightly.

***Results and Discussion***

The level of overall self-sufficiency is 56% in Switzerland, 81% in Germany and 60% in the United Kingdom. However, individual crop groups do not reach a similar level of self-sufficiency. Table S1 summarizes the differences for selected main crops in and between these countries. Wheat and potato production consistently achieve high levels of self-sufficiency (81-109%), whereas fruit self-sufficiency varies from 16% in the United Kingdom to 48 % in Austria.

**Table S1: Self-sufficiency for selected crops and overall self-sufficiency**

| **Crop Group** | **Self-sufficiency (%)** | | | |
|---|---|---|---|---|
| | **Austria** | **Switzerland** | **Germany** | **United Kingdom** |
| Cereals | 94 | 81 [2] | 109 | 100 |
| Potatoes | 90 | 90 [2] | 150 | 70 |
| Fruits | 48 | 21 | 20 | 16 |
| Vegetables | 58 | 39 | 38 | 50 |
| Pulses | 81 | 3 | 61 | - |
| Overall | - [1] | 56 | 81 | 60 |
| | BML (2023), Statistik Austria (2023) | BLW (2023), BFS (2022), SBV (2023) | BLE (n.d.-a) Statista (2024) | DEFRA (2023), Statista Research Department (2021) |

[1] No data available on overall self-sufficiency, only for individual plant or animal products.
[2] Bread wheat and table potatoes
Different references and harvest years may show deviations for same crop

The percentage of organic farming area in Austria, Switzerland, Germany and United Kingdom are 26%, 17%, 10% and 3%, respectively (Statista, 2023). Brückler et al. (2018) analyzed the yields of organic and conventional farms in detail by comparing the differences in yields per crop in one region with differences in yields in other regions. For example, the organic wheat yields in Germany and the United Kingdom are 46 to 47 % of conventional wheat yields (see Table S2), in Austria and Switzerland organic yields reach 66 % and 72 % of conventional production, respectively. Brückler et al. (2018) explained these differences

with the different productivity levels in the various regions: They concluded that the yield differences between conventional and organic farms are greater at higher productivity levels and refer to countries with large particularly fertile regions such as the United Kingdom (with the English Lowlands) or Germany (Gabriel et al., 2013; Noleppa, 2016; Ströbel, 2024).

**Table S2: Relative Yield Organic versus Conventional**

| Crop Group | Relative Yield Organic versus Conventional (%) | | | | |
|---|---|---|---|---|---|
| | **Austria** | **Switzerland** | | **Germany** | **United Kingdom** |
| Wheat | 64 | 90 | 72 | 47 | 46 |
| Potatoes | 51 | 58-66 | 69 | 50-60 | - |
| All Crops | - | 80 | - | 48-51 | - |
| | Brückler et al. (2018) | Mäder et al. (2002) (plot data) | FiBL (2023) (farm data) | BLE (n.d.-b, n.d.-c), Noleppa (2016) Ströbel (2024) | Gabriel et al. (2013) |

Different references and harvest years may show deviations for same crop.
Non-marketable crops in organic rotations were not accounted for.

Smith et al. (2019) predict what would happen in England and Wales if 100% of the food needed to feed the population is produced on organic farms. They estimate that this conversion would result in a drop in total food production and production of human edible protein by about 40% compared to the conventional farming baseline. Smith et al. (2019) noted “The decrease is due to the smaller crop yields per unit of land area under organic management and the need to introduce fertility-building grass leys with nitrogen-fixing legumes within crop rotations.” They point out “The latter requirement is a farming system-level effect that is not captured in crop-level comparisons”. Brückler et al. (2018) make a similar comment: "If we account for the non-marketable area within a crop rotation, the crop yields may be even lower." They estimate from a survey conducted in Austria that non-marketable crops such as clover and alfalfa are grown on 16% of the area of an organic crop rotation.

Therefore, projections of the effects of changes in production methods (organic vs conventional) should be based on surveys at farm level including typical crop rotations for the respective farming system. Yield comparisons derived from small plot trials and without considering the proportion of non-marketable/non-edible crops in a rotation may not reflect the actual production capacities. Furthermore, on a country level growing regions with different fertility/production level need to be adequately represented for yield estimates.

Reducing the consumption of animal products could be an option to reduce dependence on (land) imports. If this path is taken, it should be borne in mind that more vegetables, especially pulses as a human edible protein source and fruits should be grown domestically in order to provide the population with a more complete, healthy and balanced diet from regional production. However, the level of self-sufficiency for vegetables and fruits is already rather low (Table S1). The conditions for the production of such essential crops need to be monitored very closely to avoid a greater shift to non-domestic production or a collapse of the fragile domestic production putting the security of food supply at risk.

In this context, the advisory service of the German federal state of Rhineland-Palatinate notes strong concerns that the cultivation of marketable fruit is becoming increasingly difficult because fewer products are available to combat diseases in demanding specialty crops: According to Harzer (2022), "the requirement to reduce the use of chemical pesticides in the EU by 50% by 2030 would lead to a reduction in harvests of at least 30%. Many farmers would no longer be economically viable, and their existence would be threatened". The number of approved active substances in the EU fell by 61 to 231 (-21%) active substances between 2019 and 2022. At the same time, the development of biopesticides is stagnating.

### ***Conclusion***

Plans for conversion from conventional to organic production need to consider differences in self-sufficiency levels for individual crops and differences in fertility/production levels between growing regions for valid yield estimates. By this means realistic projections can be made about the amount of food that will be maintained in local production, in addition to the gains in biodiversity, when food is imported from abroad.

### ***References***

BFS (Bundesamt für Statistik) (2022). Legislaturindikator: Selbstversorgungsgrad bei Nahrungsmitteln. BFS, CH-2010 Neuchatel, Switzerland. Retrieved August 08, 2023 https://www.bfs.admin.ch/bfs/de/home/statistiken/querschnittsthemen/monitoring-legislaturplanung/alle-indikatoren/leitline-3-sicherheit/selbstversorgungsgrad.html

BLE (Bundesanstalt für Landwirtschaft und Ernährung). (n.d.-a). *Der Selbstversorgungsgrad mit Lebensmitteln in Deutschland: BZL*. https://www.landwirtschaft.de/wirtschaft/agrarmaerkte/markt-und-versorgung/der-selbstversorgungsgrad-wie-ist-es-in-deutschland-um-die-versorgung-mit-lebensmitteln-bestellt

BLE (Bundesanstalt für Landwirtschaft und Ernährung). (n.d.-b). *Ökologischer Kartoffelanbau*. Retrieved August 16, 2024, from https://www.oekolandbau.de/landwirtschaft/oekologischer-pflanzenbau/spezieller-pflanzenbau/ackerbau/hackfruechte/oekologischer-kartoffelanbau/#:~:text=Wegen%20der%20besonders%20umweltschonenden%20Produktionsbedingungen%20werden%20im%20%C3%96kolandbau,damit%2050-60%20Prozent%20des%20Niveaus%20im%20konventionellen%20Anbau

BLE (Bundesanstalt für Landwirtschaft und Ernährung) (Ed.). (n.d.-c). *Vergleich: Erträge im ökologischen und konventionellen Landbau (BZL-Infografik)*. Retrieved August 16, 2024, from https://www.oekolandbau.de/vergleich-ertraege-im-oekologischen-und-konventionellen-landbau-bzl-infografik/

BLW (Bundesamt für Landwirtschaft) (2023). Agrarbericht 2023. Selbstversorgungsgrad. CH-3003 Bern, Switzerland. Retrieved December 18, 2023. https://www.agrarbericht.ch/de/markt/marktentwicklungen/selbstversorgungsgrad

BML (Bundesministerium für Landwirtschaft Forstwirtschaft, Regionen & Wasserwirtschaft) (2023). Selbstversorgungsgrad bei Lebensmitteln. Vienna, Austria. Retrieved December 18, 2023. https://info.bml.gv.at/themen/lebensmittel/lebensmittel-in-oesterreich/selbstversorgungsgrad.html

Brückler, M., Resl, T., & Reindl, A. (2018). Comparison of organic and conventional crop yields in Austria. Die Bodenkultur: Journal of Land Management, Food and Environment, 68(4), 223-236. https://sciendo.com/article/10.1515/boku-2017-0018

DEFRA (Department for Environment, Food and Rural Affairs) (2023, Oct 05). United Kingdom Food Security Report 2021. Retrieved December 18, 2023. https://www.gov.uk/government/statistics/united-kingdom-food-security-report-2021/united-kingdom-food-security-report-2021-theme-2-uk-food-supply-sources

FiBL (Forschungsinstitut für den Biologischen Landbau) (2023). Bio: Produktionskosten, Erträge und Preise. Retrieved 2023, December 20. https://www.bioaktuell.ch/grundlagen/oekonomie/bio-produktionskosten-ertraege-und-preise

Gabriel, D., Sait, S. M., Kunin, W. E., & Benton, T. G. (2013). Food production vs. biodiversity: comparing organic and conventional agriculture. Journal of applied

ecology, 50(2), 355-364. https://besjournals.onlinelibrary.wiley.com/doi/epdf/10.1111/1365-2664.12035

Harzer, U. (2022). Quo vadis Chemischer Pflanzenschutz. Obstbau 12/2022, 701-707. https://www.obstbau.org/rubriken/pflanzenschutz/quo-vadis-chemischer-pflanzenschutz.html

Mäder, P., Fliessbach, A., Dubois, D., Gunst, L., Fried, P., & Niggli, U. (2002). Soil fertility and biodiversity in organic farming. Science, 296(5573), 1694-1697. https://www.science.org/doi/epdf/10.1126/science.1071148

Noleppa, S. (2016). Plant protection in Germany: effects of conventional agriculture on regional and global biodiversity. HFFA Research GmbH, 04. 43 pp. Berlin, Germany. https://hffa-research.com/wp-content/uploads/2023/08/HFFA-RP-2016-04.pdf

SBV (Schweizer Bauernverband) (2023). Fokus Digital. Was isst die 9 Millionen Schweiz? Kapitel 3. Aktuelle Versorgungslage. Selbstversorgungsgrad. Retrieved December 18, 2023. https://fokus.sbv-usp.ch/ernaehrung/de/inhalt/aktuelle-versorgungslage/selbstversorgungsgrad.html

Smith, L.C., Kirk, G.J.D., Jones, P., & Williams, A.C. (2019). The greenhouse gas impacts of converting food production in England and Wales to organic methods. *Nature Communications*, *10*(1). https://doi.org/10.1038/s41467-019-12622-7

Statistik Austria (2023). Versorgungsbilanzen für pflanzliche Produkte 2021/22. Retrieved December 18, 2023. https://www.statistik.at/fileadmin/user_upload/SB_1-27_Versorgungsbilanz-pflanzl_2021_22.pdf

Statista Research Department (2021, April 13). Food production to supply ratio of the United Kingdom (UK) from 1988 to 2020. Retrieved December 18, 2023. https://www.statista.com/statistics/1119406/food-production-to-supply-ratio-in-the-united-kingdom-uk/

Statista (2023). Anteil der Bio-Anbaufläche an der landwirtschaftlichen Nutzfläche in Europa nach Ländern in den Jahren 2020 und 2021. Retrieved December 18, 2023. https://de.statista.com/statistik/daten/studie/5423/umfrage/anteil-der-oeko-flaeche-an-der-landwirtschaft-in-den-eu-2

Statista (2024). Selbstversorgungsgrad bei Hülsenfrüchten in Deutschland in den Jahren von 2012/14 bis 2021/22. Retrieved February 20, 2024. https://de.statista.com/statistik/daten/studie/76627/umfrage/selbstversorgungsgrad-in-deutschland-mit-huelsenfruechten/

Ströbel, H. (2024). Is More Organic Farming a Responsible Strategy? An Appeal for Responsible (Sustainably Intensive) Agriculture. Sustainability 16 (10), 4114. Retrieved May 20, 2024. https://doi.org/10.3390/su16104114

## 2. Methods of the prediction of long-term behavior in soil

The purpose of this Supporting Information is to provide examples on how the long-term behavior of compounds in soil is typically addressed in exposure assessments for regulatory decision making.

### *Introduction*

The long-term fate and accumulation of compounds in soil e.g. for the terrestrial ecotoxicological risk assessment needs to be addressed as stipulated by the current EU regulatory framework for pesticide authorization. The soil behavior can be either evaluated by monitoring or modelling studies.

### *Method*

Over a period of several years repeated annual applications of the test substances are performed until the final endpoint of a stationary and stable plateau concentration had been reached (Ernst et al., 2022; Sur et al., 2014).

### *Results*

The accumulation of the fungicide Bixafen in soil is shown as first example in Figure S1. The comparison of retrospective monitoring data with the modelled predictions based on EFSA approved compound data shows an excellent agreement between observations and predictions. Figure S2 provides another example of the prospective monitoring of the fungicide BCS 01 in soil until the plateau level in soil was reached after eight years (Sur et al., 2014). The soil degradation half-life in the field was optimized and amounted to 184 days (158 d – 213 d, 95% confidence interval), which was significantly smaller than the laboratory derived half-life of 321 days the regulatory accumulation

calculation was based on, which again shows the conservatism of the accumulation assessment.

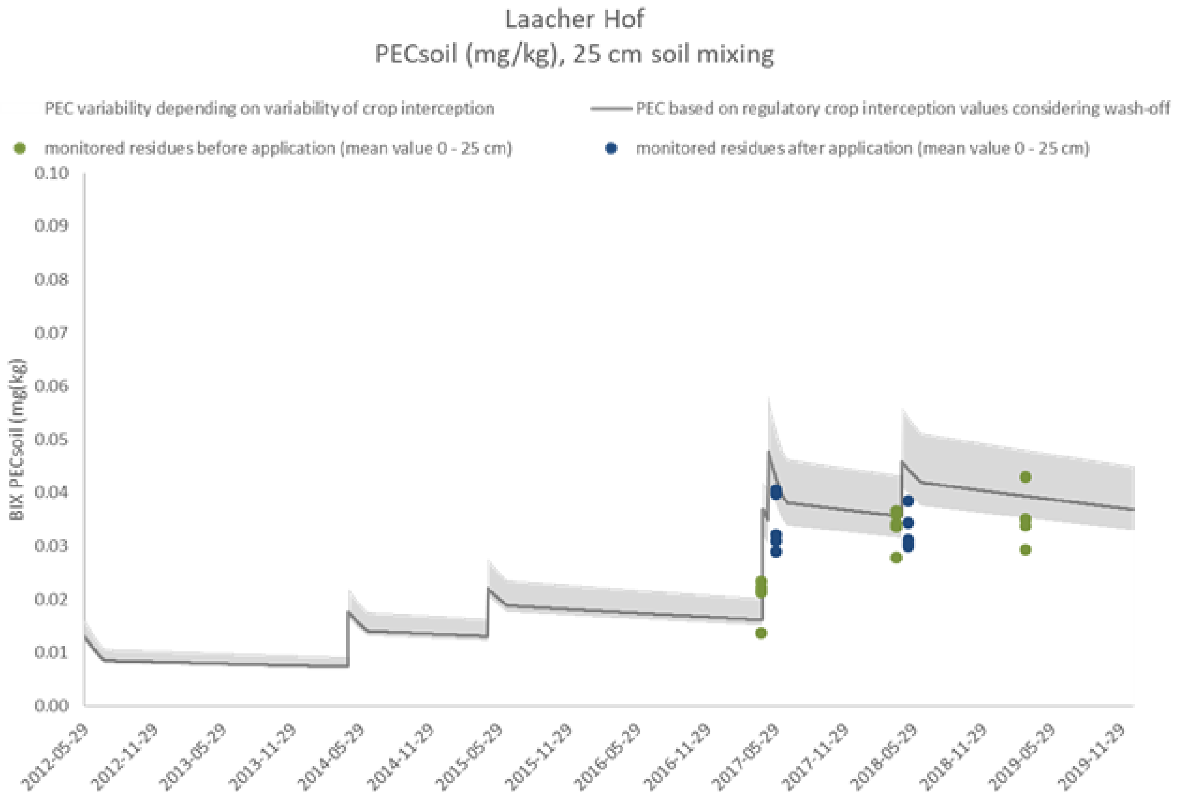


**Figure S1: Soil concentrations of Bixafen from monitoring (dots) and uncalibrated modelling (bold line with uncertainty due to canopy interception) show the protectiveness of the regulatory accumulation calculation (Ernst et al., 2022).**

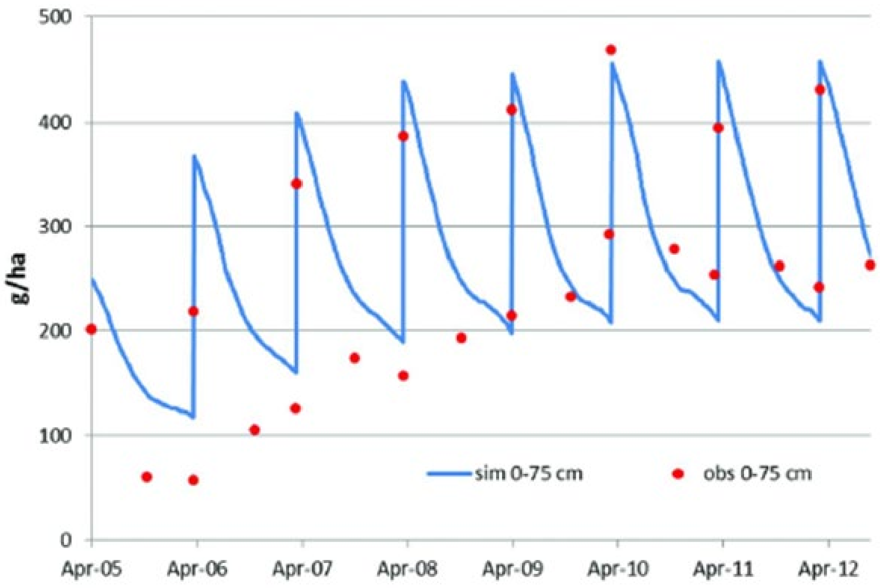


**Figure S2: Simulated vs. observed total soil residue of BCS-01 in a German terrestrial field accumulation study (entire soil profile, 0-75 cm). The optimized half-life in the field of 184 days (158 d – 213 d, 95% confidence interval) was smaller than measured in the laboratory (321 days) demonstrating the protectiveness of the laboratory-based accumulation assessment (Sur et al., 2014).**

### *Conclusion*

In summary, the long-term behavior of residues of plant protection products in the environment is typically addressed in exposure assessments for regulatory decision making. Where this is not addressed the lower-tier approaches have already provided a conservative and protective assessment of the exposure in the environment.

### *References*

Ernst, G., Agert, J., Heinemann, O., Hellpointner, E., & Gladbach, A. (2022). Realistic exposure of the fungicide bixafen in soil and its toxicity and risk to natural earthworm populations after multi-year use in cereal. *Integrated Environmental Assessment and Management*, *18*(3), 734–747. https://doi.org/10.1002/ieam.4510

Sur, R. (2014). Terrestrial field degradation based on soil, climatic, and geographic factors. In *Non-First Order Degradation and Time-Dependent Sorption of Organic Chemicals in Soil, Chapter 3* (ACS Symposium Series, Vol. 1174, pp. 39–56). American Chemical Society. https://doi.org/10.1021/bk-2014-1174.ch003.

## 3. Predicting long-term behavior in groundwater by modelling

The purpose of this Supporting Information is to explain the reasons why substances can still be detected years after their application (with decreasing concentrations). However, it is important to know that for regulatory decision making, the magnitude of the concentration of the substance is the relevant protection goal for risk assessment and not the long-term behavior of a decreasing concentration. An example is shown that simulation models can predict this long-term behavior in soil and groundwater if they are parameterized accordingly.

### *Introduction*

The regulatory leaching assessment is based on maximum predicted concentrations in shallow percolate underneath a treated field after multiple applications of the substance. This scenario for the calculation represents a worst-case. If a substance is not applied anymore, leaching of the applied substance or metabolites can still continue over a longer period of time but will always lead to lower (decreasing) concentrations. The relevant and decisive protection goal is the predicted concentration of the substance in the leachate at one meter soil depth, which is then compared to the regulatory trigger values. The magnitude of the exposure concentration at one meter depth (PECgw) following repeated annual, bi- or triannual applications of the compound over 20, 40 or 60 years is assessed as a protective surrogate of the long-term environmental exposure (EC, 2014). Such a scenario guarantees that a high (worst-case) concentration is evaluated in the assessment, as attenuation processes such as degradation, sorption and dilution, have not progressed that significantly at this depth.

An important process that can result in a retardation of leaching over a longer time period is an increase of sorption over time. Compounds can show a so-called time-dependent

sorption (TDS) behavior in soil, which is sometimes also referred to as aged sorption, kinetic sorption, long-term sorption or non-equilibrium sorption (EC, 2014; Beulke et al., 2015; Boesten, 2017). The sorption to soil particles – typically organic matter – increases with time and so does the residence time especially in the upper, carbon-rich and microbially active soil layers. This may lead to a prolonged duration of leaching to groundwater as compared to a lower-tier exposure simulation where TDS is not considered. So, TDS can describe the dynamics and the retarded entry (referred to as 'remobilization' by Schäffer et al. (2018)) of compounds into the groundwater layer more realistically.

The maximum of the predicted exposure concentration in groundwater (PECgw) will be lower with TDS as compared to exposure calculations on tier-1 level due to the slower release of the compound from the sorbed phase of the soil into the percolating porewater. Therefore, the tier-1 approach without TDS is always more protective as it is more conservative in terms of the risk assessment. TDS has been adopted by the EU Commission (EC, 2021) for the use in regulatory leaching assessments on higher tiers, which will increase the realism of exposure assessments.

Dedicated time and depth/distance profiles of the leaching behavior are not part of the regulatory exposure assessment. However, the more distant the point of evaluation (in a model or in a monitoring study) is away from the source of pesticide application, the smaller the concentrations would become and the longer it takes for a compound to reach that observation point. In addition, the assessment would also become increasingly data demanding and uncertain: Information on properties of the deeper soil and groundwater layer are in most cases not known the deeper the evaluation depth progresses. These kinds of evaluations are far from being standard and are only conducted in exceptional cases. These may include tailored research studies in drinking water catchments to predict the temporal and spatial propagation of contamination flumes, where the proper parameterization of the

hydrogeology in the models requires many additional experimental studies to gauge soil and aquifer properties at different points of the catchment under investigation. The transport of a compound from a treated field into the groundwater or to a well can easily take many years. An example for such a special study conducted in a drinking water catchment is presented below (Herrmann & Sur 2021). It should also be noted that some special cases exist, where the use of standard regulatory models and scenarios are not appropriate: These are, for example, complex substance transport pathways in Karst soils or (partially historic high) non-agricultural uses on railway tracks and industrial areas. Another case to be considered separately is the (partly pH-dependent) sorption of molecules in clay minerals if these molecules are present as cations. Undocumented applications can also be misinterpreted as retarded leaching from known authorized agricultural uses.

***Method***

Strawberry fields were treated with the fungicide tolylfluanid in a catchment between 1980 and 1990. The behavior of the non-relevant metabolite N-N-dimethylsulfamide (DMS) of tolylfluanid was investigated after the application period at various measuring points in soil and in the groundwater from the point of application downgradient to a drinking water well (Herrmann and Sur, 2021).

***Results and Discussion***

The example in Figure S3 shows the leaching of the non-relevant metabolite N-N-dimethylsulfamide (DMS) across a soil profile with a depth of 48 m. The peak concentration in porewater was measured to occur between 15 and 20 m depth in 2017. The measured porewater concentration could be well matched by applying a dual-porosity model (equivalent to TDS model), which improved the description of peak fronting and provided

more retardation of DMS in the soil profile. Again, a more simplistic, lower-tier approach would have resulted in a quicker leaching of the solute. The DMS peak is expected to enter the groundwater by 2027 (Figure S4). This example illustrates the importance of modelling and monitoring in a combined effort to predict the magnitude and duration of the exposure: A snapshot of the measured exposure was used to calibrate and parametrize the model, which then was used to predict the exposure over durations too long to be answered in a reasonable time by a monitoring study. Such approaches are extremely data hungry, costly and time consuming and are therefore only rarely conducted, e.g. in exceptional cases to manage drinking water supplies. Drinking water providers usually have a good knowledge of the hydrogeology of their catchments. Conceptually similar approaches may also be used for the interpretation of regulatory monitoring studies.

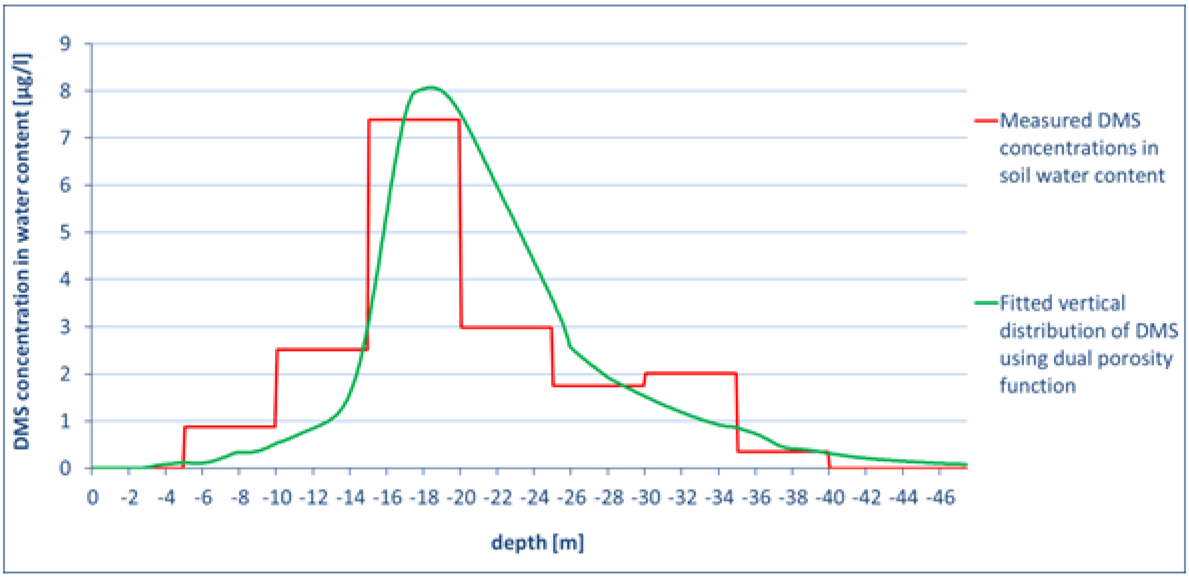


**Figure S3:** ***DMS distribution in porewater over depth calibrated to measured concentrations in a soil profile below a strawberry field treated with tolylfluanid (Herrmann and Sur, 2021).***

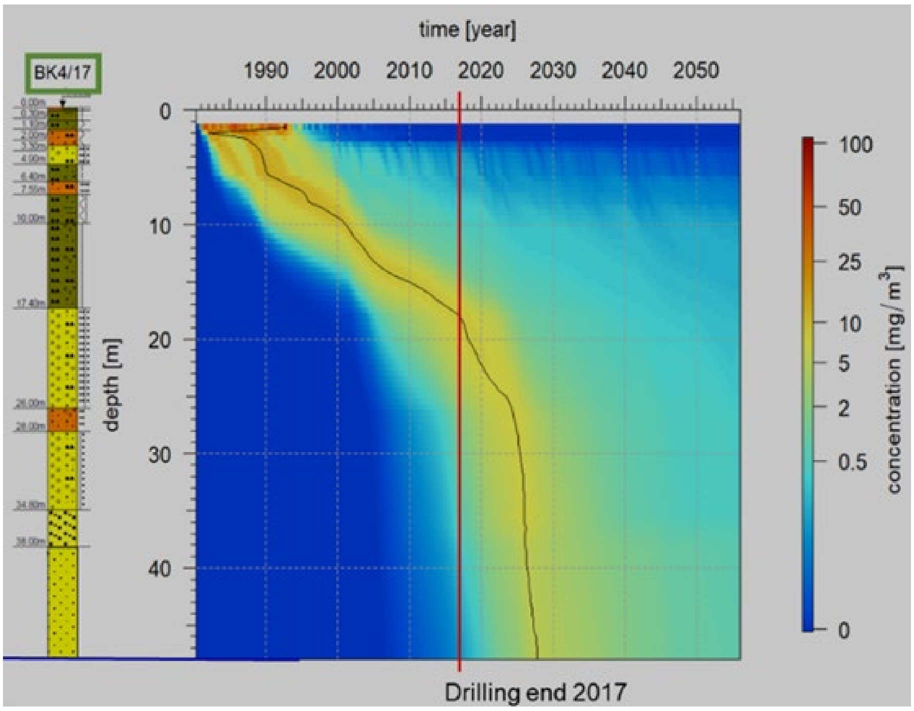


**Figure S4:** ***Simulated (HYDRUS-1D) concentrations of DMS in porewater over depth and time in a soil profile below a strawberry field treated with tolylfluanid (black curve marks maximum) (Herrmann and Sur, 2021).***

## *Conclusions*

Active substances and metabolites can still be found in water monitoring long after application of the plant protection products have happened. This may have two reasons: time-dependent sorption (TDS) leading to a slow release from soil and a large distance between source and monitoring site resulting in long travel times. However, the focus in regulatory risk assessment is on the magnitude of the exposure rather their duration. Therefore, the protection goal for the groundwater exposure assessment focusses on when highest concentrations are expected, i.e. spatially in shallow leachate underneath a treated field and temporally during a period of several repetitive applications and not at later time points. This will ensure that plant protection products are regulated today on the basis of a worst-case scenario.

***References***

Beulke, S., van Beinum, W. and Suddaby, L. (2015), Interpretation of aged sorption studies for pesticides and their use in European Union regulatory leaching assessments. Integr Environ Assess Manag, 11: 276-286. https://doi.org/10.1002/ieam.1607

Boesten, J.J.T.I. (2017). Effects of aged sorption on pesticide leaching to groundwater simulated with PEARL, Science of The Total Environment, 576: 498-507, https://doi.org/10.1016/j.scitotenv.2016.10.099.

EC (European Commission). (2014). Assessing Potential for Movement of Active Substances and their Metabolites to Ground Water in the EU. *Report of the FOCUS Ground Water Work Group, EC Document Reference Sanco/13144/2010 version 3, 613 pp.* https://esdac.jrc.ec.europa.eu/public_path/projects_data/focus/gw/NewDocs/focusGWReportOct2014.pdf

EC (European Commission). (2021). *Guidance on how aged sorption studies for pesticides should be conducted, analysed and used in regulatory assessments* (SANTE/12586/2020 – REV 1). https://food.ec.europa.eu/system/files/2022-02/pesticides_ppp_app-proc_guide_fate_aged-sorption_0.pdf

Herrmann, M., & Sur, R. (2021). Natural attenuation along subsurface flow paths based on Modelling and monitoring of a pesticide metabolite from three case studies. *Environ Sci Eur* 33, 59. https://doi.org/10.1186/s12302-021-00490-2

Schäffer, A., Filser, J., Frische, T., Gessner, M., Köck, W., Kratz, W., Liess, M., Nuppenau, E.-A., Roß-Nickoll, M., Schäfer, R., & Scheringer, M. (2018). The Silent Spring - On the need for sustainable plant protection. *Leopoldina Discussions* No. 16; 61. https://www.leopoldina.org/uploads/tx_leopublication/2018_Diskussionspapier_Pflanzenschutzmittel_EN_02.pdf

## 4. Information on markers of wastewater effluents in small surface water bodies

The purpose of this Supporting Information is to show that thorough evaluation of chemical data can provide valuable insights into the source of exposure and identify potential additional chemical stressors to be considered in improving ecological quality in surface waters.

### *Introduction*

Pesticide exposure can originate from diffuse agricultural sources (i.e. from agricultural fields treated with PPPs) or from point sources as a result of accidents or non-compliant handling of PPP (i.e. sources such as effluents from farmyards, machinery wash bays or effluents from WWTP into brooks) nor non-agricultural sources (e.g. biocides, industrial uses). Clarification of findings is required to identify actual sources and to be able to determine appropriate actions to mitigate exposure. If pesticide exposure originates from diffuse agricultural sources, the regulatory process for PPP foresees adaptation of registered uses e.g. by adding mandatory risk mitigation measures (e.g. vegetated filter strips) or use rate reductions. If pesticide exposure results from point sources or non-agricultural uses this is regulated by other pieces of legislation and requires different types of mitigation measures.

### *Method*

Neale et al. (2020) investigated 44 monitoring sites in small streams in agricultural areas in Germany. These monitoring sites are a subset of the 124 stream sites that were monitored by Liess et al. (2021) (see Supporting Information chapter 5 for details). Neale et al. (2020) analyzed water samples for pesticides and urban contaminants including several wastewater markers and recorded the presence of WWTP upstream of monitoring sites. They rated sites as “impacted” or “likely impacted” by wastewater effluents based on confirmed wastewater

treatment plants upstream and based on the detection of wastewater markers, respectively. Considered wastewater markers were 1H-Benzotriazole, 5-Methyl-1H-benzotriazole, acesulfame, acetaminophen, acetyl-sulfamethoxazole, caffeine, carbamazepine, cyclamate, diclofenac, oxypurinol, saccharin, sucralose, sulfamethoxazole and theophylline.

***Results and discussion***

Neale et al. (2020) reported that "that non-pesticide chemicals and even typical wastewater-derived chemicals were found at sites assumed to be largely free from wastewater effects prior to the study". Overall, they identified 32 sites as "impacted" or "likely impacted" by wastewater effluents (Table S3) and, therefore, it cannot be ruled out for these sites that point entries play a role in the measured concentrations of active substances. Literature shows that pesticide input via wastewater effluents is relevant. The proportion of PPPs entering surface waters from non-diffuse sources was estimated to be over 60% of the total pesticide load (Bach et al., 2005; Kokaric et al., 2023).

**Table S3: Overview of 32 out of 44 stream sections that were rated as "impacted" or "likely impacted" by wastewater effluents**

| **Sites impacted by wastewater treatment plant (WWTP) effluent** | | | | |
|---|---|---|---|---|
| **Site ID** | **Urban area in catchment [%]** | **No. of detected sewage indicators (n=14)** | **WWTP upstream (distance [km])** | **WWTP type** |
| **5** | 8.6 | 14 | Yes (2.3) | Municipal |
| **21** | 6.1 | 14 | Yes (10) | Industrial |
| **22** | 6.9 | 13 | Yes (5.7) | Industrial |
| **23** | 7.4 | 14 | Yes (1.5) | Industrial |
| **26** | 6.7 | 14 | Yes (1.8) | Municipal |
| **29** | 3.6 | 11 | Yes (2.4) | Municipal |
| **35** | 2.5 | 12 | Yes (1.4) | Municipal |
| **37** | 16.1 | 14 | Yes (5.2) | Municipal |
| **Sites likely impacted by wastewater** | | | | |
| **Site ID** | **Urban area in catchment [%]** | **No. of detected sewage indicators (n=14)** | **WWTP upstream** | **WWTP type** |
| **2** | 17.9 | 13 | No | - |
| **3** | 18.2 | 12 | No | - |
| **6** | 6.2 | 13 | No | - |
| **7** | 15.5 | 13 | No | - |
| **8** | 0.0 | 10 | No | - |
| **10** | 0.0 | 10 | No | - |
| **13** | 2.1 | 10 | No | - |
| **14** | 2.1 | 12 | No | - |
| **15** | 8.3 | 11 | No | - |
| **17** | 15.8 | 13 | No | - |
| **18** | 8.9 | 10 | No | - |
| **20** | 4.0 | 14 | No | - |
| **24** | 11.7 | 14 | No | - |
| **25** | 4.8 | 10 | No | - |
| **27** | 6.2 | 14 | No | - |
| **30** | 0.3 | 14 | No | - |
| **31** | 6.8 | 12 | No | - |
| **32** | 6.8 | 12 | No | - |
| **33** | 17.1 | 14 | No | - |
| **36** | 6.0 | 13 | No | - |
| **38** | 10.5 | 11 | No | - |
| **40** | 9.7 | 11 | No | - |
| **42** | 2.4 | 13 | No | - |
| **44** | 11.6 | 11 | No | - |

***Conclusion***

At monitoring sites with chemical exposure profiles that suggest point source inputs, the source of the contamination must be determined. If point sources are identified or are likely, mitigation measures must target these sources to be effective. An a priori assumption

that pesticide contamination is generally due to diffuse inputs is neither appropriate nor generally effective for water protection.

***References***


Bach, M., Röpke, B., & Frede, H. G. (2005). Pesticides in rivers – Assessment of source apportionment in the context of WFD. *European Water Management Online*. https://www.ewa-online.eu/tl_files/_media/content/documents_pdf/Publications/E-WAter/documents/65_2005_02.pdf

Korkaric, M., Lehto, M., Poiger, T., De Baan, L., Mathis, M., Ammann, L., Hanke, I., Balmer, M., & Blom, J. (2023). Nationale Risikoindikatoren für Pflanzenschutzmittel. *Agrocope Science*, *154*, ISSN 2296-729X. https://doi.org/10.34776/as154g

Neale, P. A., Braun, G., Brack, W., Carmona, E., Gunold, R., König, M., Krauss, M., Liebmann, L., Liess, M., Link, M., Schäfer, R. B., Schlichting, R., Schreiner, V. C., Schulze, T., Vormeier, P., Weisner, O., & Escher, B. I. (2020). Assessing the mixture effects in in vitro bioassays of chemicals occurring in small agricultural streams during rain events. *Environmental Science & Technology*, *54*(13), 8280–8290. https://doi.org/10.1021/acs.est.0c02235

## 5. Exceedances of regulatory acceptable concentrations of plant protection products in small streams in agricultural areas in Germany

The purpose of this Supporting Information is to review the chemical monitoring data from a recent surface water monitoring campaign in Germany to understand the causes for reported RAC exceedances.

### *Introduction*

A surface water monitoring campaign (Kleingewässermonitoring (KGM), 2018-2019)) was commissioned by the German Federal Environment Agency (UBA) to assess the chemical and biological condition of small agricultural watercourses and to verify the effectiveness of risk assessment and management in the pesticide approval process. At 124 selected stream sites, water samples were taken as i) regular grab samples or ii) composite samples triggered by a water level rise > 5 cm above dry weather level in the streams (event-driven samples). The samples were analyzed for 75 active substances of plant protection products (PPP)s, biocides, pharmaceuticals, industrial chemicals, and wastewater markers. The final campaign report concluded that regulatory acceptable concentrations (RAC) of active substances were exceeded in more than 73% of the investigated watercourse sections, indicating a general underestimation of the actual risk in pesticide approval (Liess et al, 2022).

### *Methods*

Monitoring data on the 75 active substances from the campaign (Liess et al., 2021) were compared with i) RAC values for these substances listed by UBA as to be relevant at the time of the monitoring (KGM-RAC) (Liess et al., 2022) and ii) RAC values for these substances

that were legally binding at the time of the latest national approval of the PPPs containing a substance (BVL-RACs) (*BVL - Zulassungsberichte*, n.d.).

***Results***

In the event-driven samples, exceedances of the KGM-RACs were observed at least once for 33 out of the 75 active substances. For approximately 75% of the 33 substances, the KGM-RACs differed from the legally binding BVL-RACs and were mostly lower due to various reasons (e.g., an additional safety factor of 3 was applied or supportive (higher-tier) studies and data from EU documents were not considered) (Table S4). The overall number of BVL-RAC exceedances in the event-driven samples was approximately 50% lower compared to the overall number of KGM-RAC exceedances (Table S5). Exceedances of the BVL-RACs were observed for 26 of the 75 active substances and for only 3 of them exceedances were observed in more than 5% of the samples (Table S5). At 74 of the 124 monitoring sites (60%), no (40%) or only one (20%) BVL-RAC exceedance was observed during the two-year monitoring period. Only at 15 sites (12%) BVL-RACs were exceeded six or more times (Figure S5).

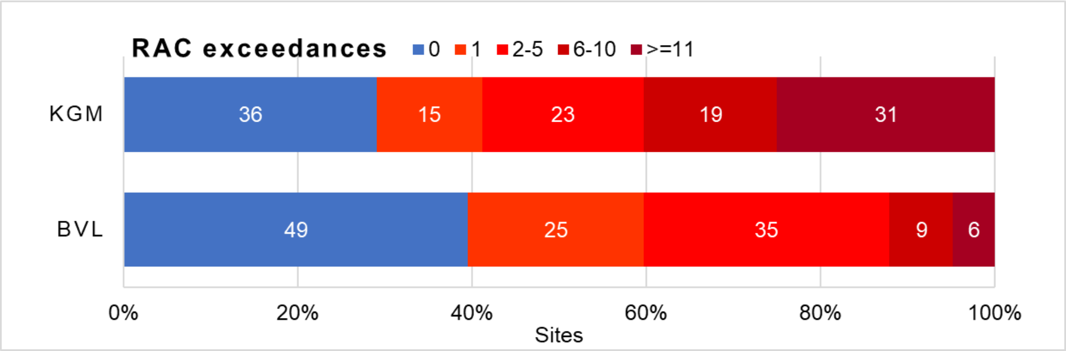


**Figure S5: RAC exceedances per site detected in event-driven samples (n = 124).**

At all sites with five or more BVL-RAC exceedances (n = 24) known wastewater markers co-occurred with the active substances. Considering a total of 14 waste water

markers (1H-benzotriazole, 5-methyl-1H-benzotriazole, acesulfame, acetaminophen, acetyl-sulfamethoxazole, caffeine, carbamazepine, cyclamate, diclofenac, oxypurinol, saccharin, sucralose, sulfamethoxazole and theophylline), 18 sites were likely impacted by waste water according to the classification applied by Neale et al. (2020) on a subset of the 124 sites (likely impacted by wastewater: 10 or more waste water markers detected) (Table S6). It is noteworthy that for Fipronil, a substance used in veterinary medicine and as a biocide, the majority of sites with RAC exceedances was likely impacted by wastewater.

**Table S4: Active substances of PPP with i) measured concentrations in event-driven samples exceeding BVL-RAC values and ii) BVL-RAC values differing from KGM-RAC values (n = 26)**

| Substance | CAS No. | KGM RAC (µg/L) | AF | BVL RAC (µg/L) | AF | BVL registration number |
|---|---|---|---|---|---|---|
| Acetamiprid | 135410-20-7 | 0.018 | 140 | 0.196 | 100 | 005655-00/25 |
| Azoxystrobin | 131860-33-8 | 0.55 | 100 | 0.95 | 3 | 008293-00/00 |
| Clothianidin | 210880-92-5 | 0.007 | 3 | 0.01 | 2 | 025495-00/00 |
| Cyazofamid | 120116-88-3 | 0.77 | 30 | 1.1 | 10 | 006860-00/01 |
| Dichlorprop-P | 15165-67-0 | 5.2 | 30 | 2.03 | 10 | 043729-00 |
| Diflufenican | 83164-33-4 | 0.025 | 10 | 0.11 | 2 | ZN1 007367-00/00 |
| Dimethenamid-P | 163515-14-8 | 1.52 | 9 | 2.4 | 5 | 024803-00/19-003 |
| Dimoxystrobin | 149961-52-4 | 0.0316 | 10 | 0.41 | 10 | 025533-00/01-001 |
| Epoxiconazole | 133855-98-8 | 0.46 | 10 | 0.43 | 10 | 006912-00/00 |
| Ethofumesate | 26225-79-6 | 15.6 | 10 | 24 | 10 | 024257-00/00 |
| Flufenacet | 142459-58-3 | 12 | 1 | 2.4 | 5 | ZN1 007367-00/00 |
| Foramsulfuron | 173159-57-4 | 0.034 | 10 | 0.095 | 4 | ZN8 007424-00/00 |
| Imidacloprid | 138261-41-3 | 0.009 | 3 | 0.6 | 5 | 024787-00/00 |
| Isopyrazam | 881685-58-1 | 0.258 | 100 | 0.287 | 10 | 008293-00/00 |
| Lenacil | 96639 | 0.35444 | 9 | 1.06 | 3 | na |
| MCPA | 94-74-6 (DMA salt: 2039-46-5) | 4.33 | 30 | 90 | 10 | 006396-00/02 |
| Mecoprop-P | 93-65-2 | 0.9 | 30 | 160 | 10 | 053678-00 |
| Metamitron | 41394-05-2 | 38 | 10 | 373.3 | 3 | 006470-00/00 |
| Nicosulfuron | 111991-09-4 | 0.09 | 10 | 0.11 | 10 | 006946-00/00 |
| Picoxystrobin | 117428-22-5 | 0.057 | 100 | 0.65 | 100 | 024658-00/02 from 2014 |
| Pirimicarb | 23103-98-2 | 0.17 | 10 | 0.09 | 10 | 062470-00 |
| S-Metolachlor | 87392-12-9 | 1.22 | 30 | 10 | 2 | 024613-00 |
| Tebuconazole | 107534-96-3 | 0.625 | 10 | 0.578 | 10 | 006855-00/00 |
| Terbuthylazine | 5915-41-3 | 1.9 | 10 | 3.3 | 3 | 005692-00 |
| Thiacloprid | 111988-49-9 | 0.004 | 3 | 0.31 | 5 | 024714-00/00 |
| Thiamethoxam | 153719-23-4 | 0.043 | 3 | 0.265 | 20 | 006212-00 |

**Table S5: Active substances of PPP with measured concentrations exceeding KGM-RAC values in more than ten event-driven samples - percentage of RAC exceedances considering KGM-RAC and BVL-RAC values**

| Substance | KGM-RAC exceedance | % of EDS samples | BVL-RAC exceedance | % of EDS samples |
|---|---|---|---|---|
| Thiacloprid [a] | 117 | 32.32 | 4 | 1.10 |
| **Clothianidin** | **50** | **13.81** | **41** | **11.33** |
| **Fipronil** [b] | **43** | **11.88** | **43** | **11.88** |
| **Methiocarb** [c] | **32** | **8.84** | **32** | **8.84** |
| Imidacloprid | 31 | 8.56 | 0 | 0.00 |
| Nicosulfuron | 18 | 4.97 | 15 | 4.14 |
| Terbuthylazine | 18 | 4.97 | 14 | 3.87 |
| Lenacil | 17 | 4.70 | 7 | 1.93 |
| Diflufenican | 14 | 3.87 | 1 | 0.28 |
| Thiamethoxam | 13 | 3.59 | 4 | 1.10 |
| Foramsulfuron | 12 | 3.31 | 8 | 2.21 |
| S-Metolachlor | 12 | 3.31 | 4 | 1.10 |
| Acetamiprid | 10 | 2.76 | 2 | 0.55 |
| Dimethenamid-P | 10 | 2.76 | 6 | 1.66 |
| **Total no. of RAC exceedances considering all substances** | **460** | **./.** | **225** | **./.** |

[a] also used as a biocide in wood protection
[b] used in veterinary medicine, and as a biocide, authorization for agricultural use expired in 2017
[c] also used as a snail bait in the household and garden sector, or as a biocide in insect sprays together with Thiacloprid

**Table S6: KGM sampling sites with five and more BVL-RAC exceedances (n = 24) – Co-occurrence of RAC exceedances and wastewater markers (Wastewater treatment plant: WWTP)**

| | | No. of samples with BVL-RAC exceedances during the monitoring period 2018/2019 | | | | No. of detected wastewater markers (n = 14) [a] | Maximum concentration of selected urban contaminants during the monitoring period 2018/2019 [µg/L] | | | |
|---|---|---|---|---|---|---|---|---|---|---|
| **Site** | **WWTP upstream** | **Event-driven samples** | **Grab samples** | **Total** | **Substances** | | **Saccharin** [a, e] | **Metformin** [b] | **Oxypurinol** [c] | **2-BTSA** [d] |
| S14 | yes | 5 | 0 | 5 | Clothianidin (1), Dimethenamid-P (1), Foramsulfuron (2), Nicosulfuron (1) | 12 | 0.8 | 3.6 | 4.1 | 0.6 |
| S31 | | 3 | 2 | 5 | **Fipronil** (1), Thiacloprid (4) | 7 | 0.6 | 0.1 | 4.7 | 1.6 |
| S40 | | 3 | 2 | 5 | Clothianidin (4), Pirimicarb (1) | 9 | 2.9 | 0.0 | 0.0 | 1.4 |
| S41 | | 5 | 0 | 5 | Bromoxynil (1), Epoxiconazole (1), Mesotrione (1), Methiocarb (1), Terbuthylazine (1) | 10 | 12.0 | 0.3 | 0.0 | 0.4 |
| S47 | yes | 3 | 2 | 5 | **Fipronil** (2), Foramsulfuron (2), Methiocarb (1) | 12 | 9.5 | 5.6 | 0.0 | 1.1 |
| S56 | | 3 | 2 | 5 | **Fipronil** (5) | 13 | 1.5 | 10.4 | 2.7 | 1.8 |
| S67 | | 5 | 0 | 5 | Clothianidin (1), Foramsulfuron (1), Mesotrione (1), Methiocarb (1), Nicosulfuron (1) | 13 | 0.7 | 2.8 | 27.4 | 0.8 |
| S74 | | 4 | 1 | 5 | Clothianidin (1), **Fipronil** (1), Methiocarb (3) | 13 | 2.2 | 0.8 | 0.0 | 0.9 |
| S80 | | 5 | 0 | 5 | Clothianidin (1), Mesotrione (1), S-Metolachlor (1) | 10 | 0.6 | 0.6 | 5.2 | 1.6 |
| S10 | yes | 2 | 4 | 6 | **Fipronil** (4), Mesotrione (1), Nicosulfuron (1) | 13 | 0.7 | 2.4 | 29.2 | 0.8 |
| S25 | | 6 | 0 | 6 | Clothianidin (1), Dimethenamid-P (1), Foramsulfuron (1), Lenacil (1), Methiocarb (1), Terbuthylazine (1) | 10 | 0.8 | 0.4 | 0.0 | 1.3 |
| S39 | | 3 | 3 | 6 | Clothianidin (2), Lenacil (2), Methiocarb (1), Pirimicarb (1) | 10 | 2.7 | 4.1 | 1.9 | 1.9 |
| S32 | | 6 | 2 | 8 | Clothianidin (5), **Fipronil** (1), Thiamethoxam (2) | 8 | 1.4 | 0.0 | 0.0 | 1.6 |
| S55 | | 4 | 4 | 8 | Clothianidin (1), **Fipronil** (6), Methiocarb (1) | 14 | 1.7 | 12.1 | 2.9 | 4.5 |
| S60 | | 2 | 6 | 8 | **Fipronil** (5), Imidacloprid (1), Methiocarb (1) | 14 | 2.0 | 16.8 | 9.3 | 2.4 |
| S43 | | 8 | 1 | 9 | Clothianidin (3), **Fipronil** (1), Lenacil (2), Methiocarb (1), Pirimicarb (2) | 14 | 3.3 | 0.7 | 14.9 | 3.8 |
| S57 | | 3 | 7 | 10 | Azoxystrobin (1), **Fipronil** (8), Thiacloprid (1) | 14 | 9.0 | 68.1 | 40.3 | 4.6 |
| S78 | | 10 | 0 | 10 | Bromoxynil (1), Clothianidin (2), Mesotrione (1), Nicosulfuron (2), S-Metolachlor (1), Terbuthylazine (3) | 7 | 2.8 | 0.0 | 0.0 | 0.2 |
| S59 | yes | 4 | 8 | 12 | **Fipronil** (10), Methiocarb (1), Tebuconazole (1) | 14 | 3.6 | 3.9 | 10.4 | 2.4 |
| S38 | | 12 | 3 | 15 | Clothianidin (5), Lenacil (2), Methiocarb (2), Nicosulfuron (1), Pirimicarb (3), Terbuthylazine (2) | 1 | 0.0 | 0.0 | 2.6 | 0.1 |

| | | No. of samples with BVL-RAC exceedances during the monitoring period 2018/2019 | | | | No. of detected wastewater markers (n = 14) [a] | Maximum concentration of selected urban contaminants during the monitoring period 2018/2019 [µg/L] | | | |
|---|---|---|---|---|---|---|---|---|---|---|
| **Site** | **WWTP upstream** | **Event-driven samples** | **Grab samples** | **Total** | **Substances** | | **Sac-charin** [a, e] | **Met-formin** [b] | **Oxy-purinol** [c] | **2-BTSA** [d] |
| S35 | | 13 | 4 | 17 | Clothianidin (8), Flufenacet (1), Lenacil (1), Nicosulfuron (1), Pirimicarb (3), Tebuconazole (1), Terbuthylazine (1), Thiamethoxam (1) | 10 | 2.1 | 0.2 | 10.9 | 2.8 |
| S83 | | 16 | 3 | 19 | Acetamiprid (1), Bromoxynil (1), Clothianidin (5), Dimethenamid-P (1), Foramsulfuron (2), Nicosulfuron (3), Pethoxamid (3), Terbuthylazine (3) | 5 | 0.5 | 0.1 | 8.3 | 3.3 |
| S98 | | 16 | 3 | 19 | **Fipronil** (17), Methiocarb (1), Pirimicarb (1) | 14 | 1.8 | 4.0 | 55.9 | 2.5 |
| S85 | | 32 | 14 | 46 | Azoxystrobin (4), Bromoxynil (2), Clothianidin (3), Dichlorprop-P (4), Diflufenican (2), Epoxiconazole (4), **Fipronil** (4), Florasulam (1), Isopyrazam (4), Mesotrione (2), Nicosulfuron (6), Pethoxamid (2), Spiroxamine (2), Tebuconazole (1), Terbuthylazine (2), Thiacloprid (3) | 11 | 1.1 | 1.0 | 24.5 | 5.8 |

[a] 1H-Benzotriazole, 5-Methyl-1H-benzotriazole, Acesulfame, Acetaminophen, Acetyl-sulfamethoxazole, Caffeine, Carbamazepine, Cyclamate, Diclofenac, Oxypurinol, Saccharin, Sucralose, Sulfamethoxazole, Theophyllin

[b] Oxypurinol: pharmaceutical metabolite of the antigout pharmaceutical allopurinol

[c] Metformin: Pharmaceutical

[d] 2-Benzo-thiazolesulfonic acid: Rubber production, street runoff marker

[e] marker for untreated wastewater, would be degraded if treated in a WWTP (Neale et al., 2020)

### *Conclusion*

In conclusion, the results of the re-evaluation of the campaign data suggests that surface water exposure under real world conditions is below levels of concern for the vast majority of analyzed active substances and sites, indicating proper functioning of the pesticide approval process. For sites with measured concentrations above levels of concern the impact of agricultural point sources and non-agricultural sources needs to be clarified to be able to decide on appropriate mitigation measures.

### *References*


*BVL - Zulassungsberichte*. (n.d.). https://www.bvl.bund.de/DE/Arbeitsbereiche/04_Pflanzenschutzmittel/01_Aufgaben/02_ZulassungPSM/02_Zulassungsberichte/psm_zulassungsberichte_node.html

Liess, M., Liebmann, L., Lück, M., Vormeier, P., Weisner, O., Foit, K., Knillmann, S., Schäfer, R. B., Schulze, T., Krauss, M., Brack, W., Reemtsma, T., Halbach, K., Link, M., Schreiner, V.C., Schneeweiss, A., Möder, M., Weitere, M., Kaske, O., von Tümpling, W., Gunold, R., Ulrich, N., Paschke, A., Schüürmann, G., Schmitt Jansen, M., Küster, E., & Borchardt, D. (2022). Umsetzung des Nationalen Aktionsplans zur nachhaltigen Anwendung von Pflanzenschutzmitteln (NAP) – Pilotstudie zur Ermittlung der Belastung von Kleingewässern in der Agrarlandschaft mit Pflanzenschutzmittel-Rückständen. *UBA Texte 07/2022*, 319 pp. https://www.umweltbundesamt.de/publikationen/umsetzung-des-nationalen-aktionsplans-zur-1

Liess, M., Liebmann, L., Vormeier, P., Weisner, O., Altenburger, R., Borchardt, D., Brack, W., Chatzinotas, A., Escher, B. I., Foit, K., Gunold, R., Henz, S., Hitzfeld, K. L., Schmitt-Jansen, M., Kamjunke, N., Kaske, O., Knillmann, S., Krauß, M., Küster, E., .

. . Reemtsma, T. (2021). *The lowland stream monitoring dataset (KgM, Kleingewässer-Monitoring) 2018, 2019* [Dataset]. https://doi.org/10.1594/pangaea.931673

Neale, P. A., Braun, G., Brack, W., Carmona, E., Gunold, R., König, M., Krauss, M., Liebmann, L., Liess, M., Link, M., Schäfer, R. B., Schlichting, R., Schreiner, V. C., Schulze, T., Vormeier, P., Weisner, O., & Escher, B. I. (2020). Assessing the mixture effects in in vitro bioassays of chemicals occurring in small agricultural streams during rain events. *Environmental Science & Technology*, *54*(13), 8280–8290. https://doi.org/10.1021/acs.est.0c02235

## 6. Contribution of non-agricultural uses to long-term detectability of PPP residues in groundwater

The purpose of this Supporting Information is to show that relevant amounts of PPP such as atrazine may have been applied on railway tracks and industrial areas and not only on maize fields. This additional information on non-agricultural uses and entry paths of atrazine should enable a more complete evaluation on conclusions published about the effectiveness of the current authorization system.

### *Introduction*

Vonberg al. (2014) attributed elevated atrazine findings in groundwater exclusively to the agricultural use of atrazine in maize. Three railway lines cross the sampling area or are in the immediate vicinity. The authors did not consider that herbicide applications on railway tracks or other non-agricultural land (industrial or urban areas) can also affect the groundwater in the study area. Inputs from these other sources could be a reason for the increased concentrations. There is published literature on the effects of herbicide applications on railway lines and their transport in groundwater (BT 1989, LUBW 2001, LU 2002). Therefore, the authors' conclusions regarding the regulatory exposure assessment are based on incomplete assumptions about application areas, dosages, and total application rates in the study region. There is ample evidence that atrazine may have been applied on railway tracks and industrial areas and not only on maize fields of the region. An evaluation is provided which informs about the amount of substance which was usually applied to such non-agricultural areas in the past.

### *Methods*

A landscape analysis of the study area sampled by Vonberg et al. (2014) was conducted to identify elements in the landscape where atrazine could potentially have been applied according to the registered non-agricultural and agricultural uses. ATKIS and CORINE database features were used to identify the different landscape elements. The land use characteristics were taken from CORINE 1990, which marks the last possible legal use of atrazine, and the higher accuracy of ATKIS was used to calculate the quantities where appropriate. Typical amounts of atrazine in agricultural and non-agricultural applications (BT, 1989) were considered. For non-agricultural applications, the railway lines and only one industrial area ("Oberzier" transformer station) in the region studied were selected. The quantities potentially applied to all maize fields in the area are then compared with the quantities potentially applied by non-agricultural uses.

### *Results*

The amounts of active ingredient that could be applied to the railway lines and the centrally located industrial area (Figure S6) for non-agricultural uses are about half the amount that could be applied to all maize fields in the area: A total of 115 kg of atrazine was calculated for the two non-agricultural uses, compared to 243 kg of atrazine per year on all maize fields (Table S7). Of the 115 kg, the railway lines receive 87 kg per year and the centrally located electricity substation "Oberzier" receives 28 kg per year.

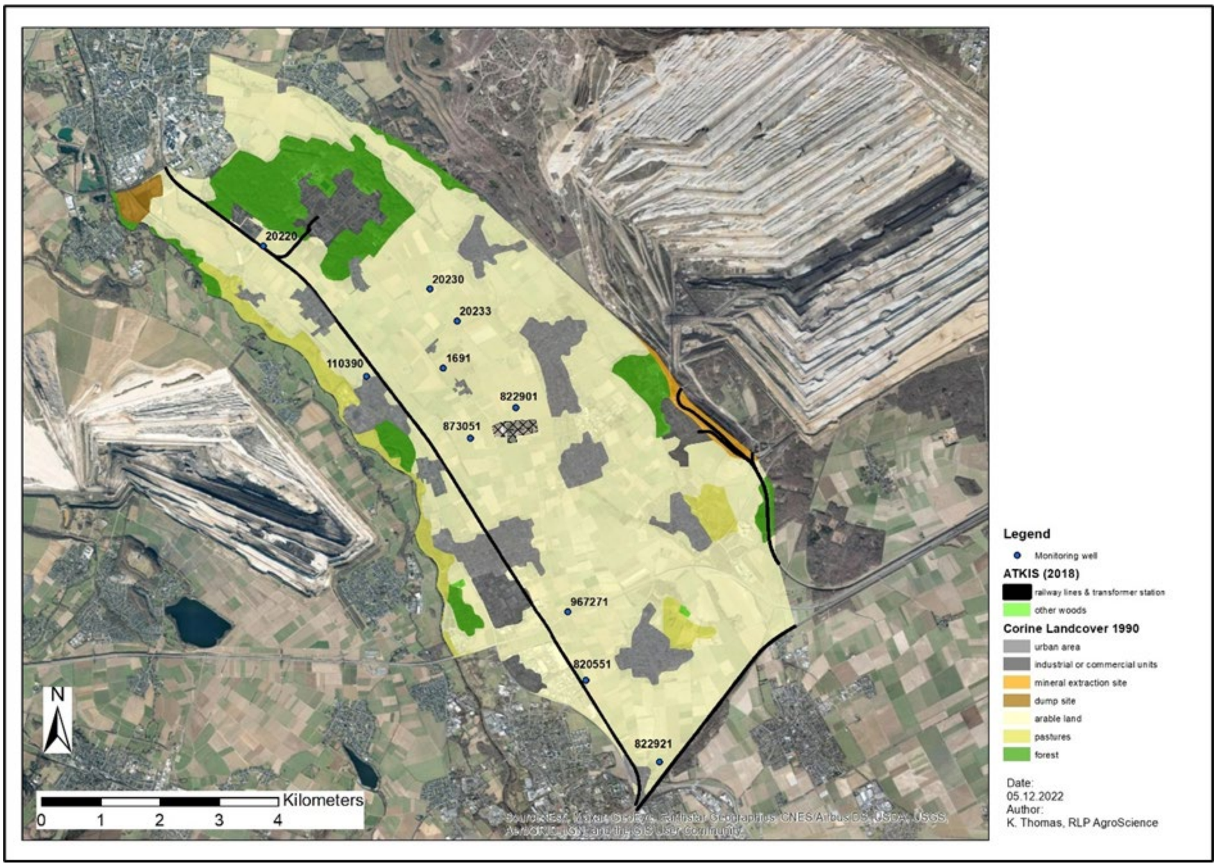


**Figure S6: Proximity of monitoring wells to areas with previous potential non-agricultural uses of atrazine (on railway lines, transformer station, industrial and urban areas). The groundwater flows in a north-westerly direction. The electricity transformer station "Oberzier" is located about 500 meters upstream to the south of monitoring well 822901.**

**Table S7: Comparison of the quantities of atrazine potentially applied to two non-agricultural landscape elements to the quantities potentially applied to maize fields. The amount of atrazine applied to railway tracks and at the Oberzier transformer station is estimated at 115 kg/year compared to estimated 243 kg/year applied on all maize fields in the sampling area.**

| Corine Land Cover No or ATKIS landscape element | Land Use Type | Area [ha] | Share of Area [%] | Mass of atrazine applied [kg/year] |
|---|---|---|---|---|
| **211: non-irrigated arable land** | Arable Land | 4052 | 69.6 | |
| **4 % fraction of area of 211** | Maize field | 162 | 2.8 | 243 |
| **ATKIS: special crop + tree nursery** | Special Crop or Tree nursery | 4 | 0.1 | |
| **231: pastures** | Pastures | 246 | 4.2 | |
| **311: broad-leaved forest** | Broad-leaved forest | 447 | 7.7 | |
| **313: mixed forest** | Mixed forest | 110 | 1.9 | |
| **112: discontinuous urban fabric** | Urban area | 508 | 8.7 | *not calculated* |
| **ATKIS: further urban areas** | Urban area | 77 | 1.3 | |
| **131: mineral extraction sites** | Coal mining area | 46 | 0.8 | |
| **132: dump sites** | Dump site | 37 | 0.6 | |
| **121: industrial or commercial units** | Industrial or commercial areas | 238 | 4.1 | *not calculated* |
| **ATKIS: further industrial or commercial unit** | Industrial or commercial areas | 15 | 0.3 | |
| **ATKIS: transformation station** | Transformation station Oberzier | 19 | 0.3 | 28 |
| **ATKIS railway** | Railway (single track) | 11 | 0.2 | |
| **ATKIS railway** | Railway (double track) | 9 | 0.2 | |
| **ATKIS railway** | Total Railway | 20 | 0.3 | 87 |
| | **Total Area** | **5818** | **100** | **358** |

Assumptions (simplified): treatment of all railway track areas and one industrial area (transformer station "Oberzier") as well as all maize fields to 100% with atrazine.
Application rate: maize areas and industrial areas 1.5 kg atrazine/ha, railway 4.35 kg atrazine/ha (8.7 kg product/ha with 50% ATR (1track: 6.6 m width, 2tracks: 12.60m; ATR load calc. accord. to BT (1989) (11/5016 (dated 28.07.1989) page 9, No. 2): product content 50% ATR of 8.2-9.2 kg active substance /ha assumed)).
Treatments on park and cemetery-like areas, sports fields, private garden areas and other industrial/commercial areas were likely at some places but are not included.
Land use features were taken from Corine 1990, which marks the last possible legal use of atrazine, and ATKIS' higher accuracy was used for calculating the sizes where appropriate

***Discussion***

Atrazine was mainly used as a maize herbicide but was also used on non-cultivated land and on railway tracks. A list of herbicides used almost exclusively in the railway track

area was published by BT in 1989. For example, atrazine, diuron and simazine were applied to railway lines in North Rhine-Westphalia over many years in several products (BT, 1989). The application rate of atrazine was also several times higher on railway lines with permeable ballast beds than on arable land. The leaching potential from treated railway tracks and transport with the groundwater flow over long distances to below agricultural land has been intensively studied (LU, 2002). Bromacil which was especially used on railway tracks (BT, 1989; LUBW, 2001) was used as a kind of tracer in order to be able to attribute the active substances found in groundwater below arable land to applications on distant railway tracks (LU, 2002). Two recent studies also show that the measuring points where threshold values were exceeded by both active substances, atrazine and bromacil, are located close to railway tracks (Castell-Exner et al., 2023, page 17, Fig. 2; LUBW, 2020, 2024, page 34).

### *Conclusions*

Considering the relevance of herbicide application on non-agricultural areas in the past for concentrations in groundwater, it is difficult to draw valid conclusions about substance behavior and existing authorization procedures, when such significant sources for input into the aquifer system of interest are omitted.

### *References*

BT (Deutscher Bundestag). (1989). *Einsatz von Pestiziden auf dem Gelände der Deutschen Bundesbahn / Use of pesticides on the premises of the German Federal Railway.* (Drucksache 11/5016). https://dserver.bundestag.de/btd/11/050/1105016.pdf

Castell-Exner, C., Danowski, A., Bauer, F., Fischer, T., Haakh, F., Laabs, V., Penning, M., Phelan, A., Peters, G., Harms, S., Resseler, H., Schmidt, C., Sturm, S., Sur, R., Winter, M. (2023). Raw Water Database of Plant Protection Products - 2022 Analysis.

BDEW, DVGW, IVA. Berlin, Bonn and Frankfurt/M., June 2023. https://tzw.de/fileadmin/user_upload/pdf/04_Projekte/Raw_Water_Database_Plant_Protection_Products_2022_Analysis.pdf.

LU (Landesamt für Umweltschutz). (2002). Pflanzenschutzmittel im Grundwasser des Saarlandes 1990 - 2000 / Plant Protection Products in the Groundwater of the Saarland 1990 - 2000. 66119 Saarbrücken, Germany.

LUBW (Landesanstalt für Umwelt Baden-Württemberg). (2001). Grundwasserüberwachungsprogramm Ergebnisse der Beprobung 2000 / Groundwater monitoring programme Results of the 2000 sampling. p. 42 - 48. Landesanstalt für Umwelt Baden-Württemberg, Karlsruhe, Germany, 108 pp.

LUBW (Landesanstalt für Umwelt Baden-Württemberg). (2020). Grundwasserüberwachungsprogramm Ergebnisse 2018 und 2019 / Groundwater monitoring programme Results of 2018 and 2019. p. 37. Landesanstalt für Umwelt Baden-Württemberg, Karlsruhe, Germany, 76 pp.

LUBW (Landesanstalt für Umwelt Baden-Württemberg). (2024). Grundwasserüberwachungsprogramm Ergebnisse der Beprobung 2022 / Groundwater monitoring programme Results of the 2022 sampling. p. 34. Landesanstalt für Umwelt Baden-Württemberg, Karlsruhe, Germany, 48 pp. https://pudi.lubw.de/detailseite/-/publication/10597

Vonberg, D., Vanderborght, J., Cremer, N., Pütz, T., Herbst, M., & Vereecken, H. (2014). 20 years of long-term atrazine monitoring in a shallow aquifer in western Germany. *Water Research*, *50*, 294–306. https://doi.org/10.1016/j.watres.2013.10.032

## 7. Monitoring for regulatory purposes in groundwater

The purpose of this Supporting Information is to provide details on the extent of a groundwater monitoring study conducted for regulatory purposes**.**

### *Introduction*

Targeted groundwater monitoring studies were requested by authorities to investigate the occurrence of the 1-2-4-H-triazole metabolite of triazole fungicides in areas of intensive use of triazole containing PPPs (BVL, 2020, 2022).

### *Methods*

Samples were taken from studies running for other active substances in cereals and oil seed rape areas or new studies were set up for sampling in sugar beet and in vegetable areas (study type described in Gimsing et al. 2019; examples II & III). Product uses were documented by surveying the farmers.

### *Results and Conclusions*

In total 295 samples were taken from 39 wells and analyzed. An overview on the results is shown in Table S8. The analytical results showed no exceedances of the regulatory threshold of 0.1 µg/L. After reviewing the monitoring data, the authority concluded that their initial concerns were adequately addressed and resolved.

**Table S8: Monitoring studies for the metabolite 1,2,4-H-triazole in groundwater**

| Crop | Monitoring wells | Samples | | | | |
|---|---|---|---|---|---|---|
| | | Total no. | <LOD[1] | <LOQ[2] | >LOQ[2] | >0.1 µg/L |
| Cereals / Oilseed rape (Study 1) | 15 | 97 | 61 | 34 | 2 | 0 |
| Cereals / Oilseed rape (Study 2[3]) | 9 | 72 | 30 | 39 | 3 | 0 |
| Cereal s/Sugar Beets | 11 | 62 | 25 | 24 | 13 | 0 |
| Vegetables | 8 | 64 | 40 | 59 | 5 | 0 |

[1] LOD: Limit of detection 0.013 µg/L
[2] LOQ: limit of quantification 0.05 µg/L.
[3] A total of 20 wells were investigated in the cereals / oilseed rape studies; four wells of study 2 were already included in study 1.

***References***

BVL (Bundesamt für Verbraucherschutz und Lebensmittelsicherheit). (2020). Appendix 1 Lists of data considered in support of the evaluation. In *Registration Report. Product code: BAS 752 03 F. Product name(s): Revytrex. Chemical active substance(s): Mefentrifluconazole, 66.7 g/L Fluxapyroxad, 66.7 g/L. Central Zone. Zonal Rapporteur Member State: Germany. NATIONAL ASSESSMENT Germany. Applicant: BASF. Submission date: 14/08/2018. MS Finalisation date: 06.02.2020 (updated document)* (pp. 640–641). https://www.bvl.bund.de/SharedDocs/Downloads/04_Pflanzenschutzmittel/01_zulassungsberichte/00A272-00-00.pdf?__blob=publicationFile&v=2

BVL (Bundesamt für Verbraucherschutz und Lebensmittelsicherheit). (2022). Appendix 1 List of data submitted by the applicant and relied on. In *Registration Report. Product code: A18253A. Product name(s): Amistar Gold. Chemical active substance(s): Azoxystrobin, 125 g/L Difenoconazole, 125 g/L. Central Zone. Zonal Rapporteur Member State: Germany. NATIONAL ASSESSMENT Germany. Applicant: Syngenta*

*Agro GmbH. Submission date: 23/04/2019. MS Finalisation date: 23/06/2022* (pp. 814–815). https://www.bvl.bund.de/SharedDocs/Downloads/04_Pflanzenschutzmittel/01_zulassungsberichte/008267-00-03.pdf?__blob=publicationFile&v=2

Gimsing, A. L., Agert, J., Baran, N., Boivin, A., Ferrari, F., Gibson, R. L., Hammond, L. A., Hegler, F., Jones, R. L., König, W., Kreuger, J., Van Der Linden, T., Liss, D., Loiseau, L., Massey, A., Miles, B., Monrozies, L., Newcombe, A., Poot, A., . . . Ulrich, U. (2019). Conducting groundwater monitoring studies in Europe for pesticide active substances and their metabolites in the context of Regulation (EC) 1107/2009. *Journal of Consumer Protection and Food Safety*, *14*(S1), 1–93. https://doi.org/10.1007/s00003-019-01211-x

## 8. Monitoring for regulatory purposes in surface water after runoff events

The purpose of this Supporting Information is to provide details on a surface water monitoring study conducted for regulatory purposes.

### *Introduction*

On request of the regulatory authorities the effectiveness of vegetated buffer strips to protect adjacent streams from runoff was investigated.

### *Methods*

The substance concentrations of terbuthylazine and desethyl-terbuthylazine in the runoff receiving streams were investigated with automatic samplers at four sites over four years (Bischoff et al., 2003; EFSA, 2011; EFSA et al., 2017a, b). Vegetated buffer strips of 10 m width had been installed at the fields adjacent to the streams at the four sites Adenstedt, Sueplingen, Ramholz and Kemading. The use of maize herbicides by farmers was documented. When the magnitude of the rainfall event triggered the sampling process, event sampling was performed after 1, 3, 6 and 9 hours in the streams. Each stream was sampled by two automatic samplers positioned upstream and downstream of the vegetated buffer zone.

### *Results and Conclusions*

The arithmetic average concentration per event was below 0.2 µg/L in the stream at the lower end of the zone with the 10 m vegetated buffer strip between fields and adjacent stream (Table S9). The average rainfall per event was 16 mm. The 90th percentile concentration in the streams was calculated to be below 0.3 µg/L after the runoff events. The effectiveness of the buffer strip installation to reduce runoff from the treated fields into the streams was confirmed.

**Table S9: Overview of terbuthylazine (TBA) and desethyl-terbuthylazine (D-TBA) concentrations in streams due to run-off events at four sites with vegetated buffer strips (n=11 events in 4 years, 1999-2002, buffer strip width: 10 metres)**

| Study Site | Date of event | Rainfall maximum [mm $d^{-1}$] | Runoff-induced in-stream concentration per rainfall event [µg $L^{-1}$] | |
|---|---|---|---|---|
| Adenstedt | June 2001 | 10 | D-TBA | < 0.1 |
| Adenstedt | June 2001 | 20 | TBA | < 0.3 |
| Adenstedt | June 2002 | 12 | TBA | < 0.1 |
| Adenstedt | June 2002 | No rainfall measured | TBA | < 0.1 |
| Adenstedt | June 2002 | 12 | TBA | < 0.1 |
| Adenstedt | July 2002 | 17 | TBA | < 0.2 |
| Sueplingen | June 1999 | 23 | TBA | < 0.1 |
| Sueplingen | June 2002 | 30 | TBA | < 0.2 |
| Ramholz | June 2002 | 22 | TBA | < 0.8 |
| Kemading | August 1999 | 20 | TBA | < 0.1 |
| Kemading | June 2001 | 14 | TBA | < 0.2 |
| **Average per event (1999-2002)** | | **16** | **TBA /D-TBA** | **< 0.2** |

Rounded values are used.

### *References*


Bischoff, G., Pestemer, W., Rodemann, B., & Küchler, T. (2003). Monitoring of terbuthylazine in surface waters adjacent to maize fields with potential run-off to prove the efficacy of vegetated buffer zones - test sites in Northern Germany. In *Pesticide in air, plant, soil & water system. Proceedings of the XII Symposium Pesticide Chemistry, Piacenza, Italy, 4-6 June 2003* (pp. 841-848). La Goliardica Pavese s.r.l., Pavia, Italy. https://www.cabdirect.org/cabdirect/abstract/20033134682.

EFSA (European Food Safety Authority). (2011). Conclusion on the peer review of the pesticide risk assessment of the active substance terbuthylazine. *EFSA Journal*, *9*(1), 1969. https://efsa.onlinelibrary.wiley.com/doi/epdf/10.2903/j.efsa.2011.1969

EFSA (European Food Safety Authority), Brancato, A., Brocca, D., Bura, L., Chiusolo, A., Marques, D. C., Crivellente, F., De Lentdecker, C., De Maglie, M., Egsmose, M., Erdos, Z., Fait, G., Ferreira, L., Goumenou, M., Greco, L., Istace, F., Jarrah, S.,

Kardassi, D., Leuschner, R., . . . Villamar-Bouza, L. (2017a). Conclusion on Pesticides Peer Review: Peer review of the pesticide risk assessment of the active substance terbuthylazine in light of confirmatory data submitted: Appendix A – List of end points for the active substance and the representative formulation. *EFSA Journal, 15*(6), e04868. https://efsa.onlinelibrary.wiley.com/action/downloadSupplement?doi=10.2903%2Fj.efsa.2017.4868&file=efs24868-sup-0001-Appendix_A.pdf

EFSA (European Food Safety Authority), Brancato, A., Brocca, D., Bura, L., Chiusolo, A., Marques, D. C., Crivellente, F., De Lentdecker, C., De Maglie, M., Egsmose, M., Erdos, Z., Fait, G., Ferreira, L., Goumenou, M., Greco, L., Istace, F., Jarrah, S., Kardassi, D., Leuschner, R., . . . Villamar-Bouza, L. (2017b). Conclusion on Pesticides Peer Review: Peer review of the pesticide risk assessment for the active substance terbuthylazine in light of confirmatory data submitted. *EFSA Journal, 15*(6), e04868. https://doi.org/10.2903/j.efsa.2017.4868

## 9. Targeted promotion of biodiversity

The purpose of this Supporting Information is to provide an English language version of a document summarizing the IVA (Industrieverband Agrar e.V.) concept for promoting biodiversity in the agricultural landscape, listing goals and fields for action (IVA, 2022). The IVA represents the interests of manufacturers of inputs for sustainable crop production in Germany.

### *Introduction*

Alongside man-made climate change, the global loss of biodiversity is the greatest challenge of this century. Agriculture has a key role to play, as the loss of habitats is also linked to the agricultural activities of recent decades. Preserving and promoting biodiversity while at the same time ensuring food security is therefore one of the most pressing issues in agriculture - conceptual and networked solutions are needed.

The IVA sees itself as an important partner in agriculture with specialist expertise and presents a concept with concrete proposals for the efficient promotion of biodiversity in the agricultural landscape. We are convinced that biodiversity must be promoted in a targeted way in the natural region, considering ecological requirements, cooperative approaches and financial incentives. This overview briefly outlines the most important aspects.

### *Fields for action*

(1) Efficient biodiversity promotion: Efficient biodiversity promotion in the agricultural landscape is needed to reverse the loss of biodiversity. The aim must be to achieve a maximum gain in biodiversity in the agricultural landscape with the lowest possible loss of agricultural productivity (area efficiency).

(2) High-quality measures in the natural environment and attractive funding: Efficient biodiversity promotion is based on the following principles: (1) focus on the natural region and implementation in cooperation; (2) quality of measures before quantity; (3) sufficient incentives through appropriate funding rates; (4) respect site conditions and use synergy effects by upgrading existing structures.

(3) Toolbox of measures: The need to promote biodiversity varies from region to region and depends on the needs of the typical species in individual natural regions. Biodiversity measures must therefore be flexibly adapted and combined. Agri-environmental programs (and contractual nature conservation) should therefore contain a toolbox of measures that enables the targeted promotion of a diverse landscape structure.

(4) 10 % near-natural areas in the normal landscape: In the normal landscape, a target of 10 % should be set for landscape structure elements, fringe structures and non-productive areas in open land, with a focus on enhancing unproductive areas.

(5) Comprehensive management plans for protected areas: To ensure the positive development of biodiversity in protected areas, there is an urgent need to draw up area-wide management plans. These are developed with the involvement of local farmers and describe potentials, requirements and measures for ecological enhancement and the promotion of specific protected assets.

(6) Using technological progress: The digital transformation in agriculture and the use of high-tech environmentally and biodiversity-friendly management techniques, for example band or spot application, have the potential to make a significant contribution to securing yields and preserving biodiversity (more targeted application, leaving out non-target plants during treatment). They also reduce the risks associated with the use of plant

protection products and fertilizers. The promotion of such methods should go beyond the status quo.

(7) Expand biodiversity consulting: The selection of suitable measures must be supported by government, nature conservation and private sector advisory services for farmers and municipalities.

***Outlook***

For biodiversity measures to be implemented successfully, they must be adequately funded. This is the only way to widely establish them while enabling a productive and sustainable agriculture. The IVA discussion paper shows that it is possible to promote biodiversity effectively across the board, even with the existing agricultural budget. It is crucial that existing support measures are used efficiently and that the success factors described above are anchored in agricultural and environmental policy. To this end, the IVA is launching a dialogue with the stakeholder groups involved - politicians, authorities, the agricultural and food industry, nature conservation organizations and civil society.

***References***


IVA (Industrieverband Agrar e. V.) (Ed.). (2022). *Biodiversität gezielt fördern: Ein Diskussionsbeitrag des IVA und Vorschläge für konkrete Maßnahmen in der Agrarlandschaft*. https://www.iva.de/sites/default/files/2023-06/IVA_2201_FL_011_Biodiversitaetskonzept_20230530_WEB.pdf
